# Alternate wetting and drying irrigation at tillering stage enhances the heat tolerance of rice by increasing sucrose and cytokinin content in panicles

**DOI:** 10.3389/fpls.2025.1598652

**Published:** 2025-05-29

**Authors:** Ti Gao, Keran Xie, Qiuqian Hu, Chao Wu, Zhenmei Wang, Wencheng Wang, Dongliang Xiong, Jianliang Huang, Shaobing Peng, Kehui Cui

**Affiliations:** ^1^ National Key Laboratory of Crop Genetic Improvement, Wuhan, Hubei, China; ^2^ Key Laboratory of Crop Ecophysiology and Farming System in the Middle Reaches of the Yangtze River, Ministry of Agriculture and Rural Affairs, Wuhan, Hubei, China; ^3^ College of Plant Science and Technology, Huazhong Agricultural University, Wuhan, Hubei, China

**Keywords:** alternate wetting and drying irrigation, high temperature stress, plant hormones, panicle initiation, spikelet fertility, transpirational cooling, sucrose-metabolizing enzymes, rice

## Abstract

High temperature events have occurred frequently in recent years under global warming conditions. High temperature during panicle initiation (PI) poses significant negative impacts on rice grain yield. Alternate wetting and drying irrigation (AWD) is widely adopted in rice cultivation. Here, to investigate the alleviating effect of AWD on heat damage to rice (*Oryza sativa* L.), we selected four rice varieties with different high-temperature resistance (Liangyoupeijiu, IR64, Huanghuazhan, Shanyou 63), two irrigation treatments at the tillering stage including AWD and continuous flooding (CF) and two temperature treatments at the PI stage (high daytime temperature, HDT; and control temperature, CK) were applied. HDT significantly reduced the yield of all four varieties except for Shanyou 63, primarily by decreasing the spikelet fertility and spikelet number, while AWD significantly improved the two traits under HDT. Moreover, HDT reduced the photosynthetic rate, increased the starch content in leaves and stems, and decreased the transport of sucrose to panicles. AWD reduced the panicle temperature, promoted sucrose synthesis in leaves, increased sucrose content and sucrose hydrolase activity in panicles under HDT. HDT also increased the content of abscisic acid and decreased that of cytokinins (CTKs), indole-3-acetic acid (IAA), and gibberellins (GAs) in panicles under CF. AWD increased the expression of CTK biosynthesis genes (*IPT*, *CYP735A*, *LOG*) and decreased that of CTK degradation gene *CKX* in roots and panicles under HDT, while enhanced the xylem sap flow rate and contents of CTKs, IAA, and GAs in panicles. Furthermore, AWD exhibited a more pronounced alleviating effect on HDT damage in heat-sensitive varieties than in heat-tolerant varieties. In summary, AWD leads to lower panicle temperature and higher photosynthetic rate, sucrose content, and CTK level in panicles, which together enhance the heat tolerance at the PI stage, and therefore is a sustainable and feasible strategy to mitigate heat-induced yield loss in rice.

## 1 Introduction

Rice (*Oryza sativa* L.) is a primary food source for more than half of the global population. It is widely cultivated in at least 130 countries and regions as reported by FAO (2023), covering a planting area of 168 million hectares with an annual production of approximately 800 million tons. However, global warming has emerged as a serious threat to rice production worldwide. The global average temperature has increased by 1.09°C in 2011–2020 compared with that in the pre-industrial period (1850–1900), and is predicted to rise by about 1.5°C in the next two decades (IPCC, 2021). Li et al. (2021) predicted that rice yield in various regions of China would decrease by 7.49% and 10.3% under 1.5°C and 2.0°C warming scenarios, respectively. The Yangtze River basin is a key rice production region in China, and has experienced frequent large-scale extreme heat events during peak summer seasons in recent years, which have led to severe annual yield losses (Shi and Ye, 2021). Therefore, it is crucial to mitigate the adverse effects of high temperature on rice production for food supply in the scenario of global warming.

It is commonly believed that high temperature stress at all growth stages can reduce the grain yield to varying degrees in rice. High temperature at the germination stage reduces the seed viability and germination rate (Liu et al., 2019), and that at the tillering stage reduces the tiller number and biomass (Liu et al., 2018; Xu et al., 2020a). At the panicle initiation stage (PI), high temperature stress causes decreases in spikelet number per panicle, spikelet fertility, and grain weight (Wu et al., 2016; Zhao et al., 2023). Similarly, high temperature at the anthesis stage reduces spikelet fertility and grain weight (Zhang et al., 2018a, 2018b), while that at the grain filling stage diminishes seed setting rate, grain weight, and grain quality (Bahuguna et al., 2017; Wada et al., 2021).

Rice plant exhibits different heat resistance at various growth stages. Sánchez et al. (2014) identified that the critical heat stress temperatures are 40.1°C, 35.3°C, 33.1°C, 37°C, and 31.3°C for germination, tillering, panicle initiation, anthesis, and grain filling in rice, respectively. In general, compared with that at the vegetative growth stage, high-temperature stress at the reproductive growth stage can cause more severe loss, particularly at the PI stage (Arshad et al., 2017; Zhang et al., 2017), which is a crucial period for the differentiation of branches and spikelets as well as development of anthers, stigmas, and pollen. High temperature adversely affects these developmental processes, significantly reducing the number of grains per panicle, seed setting rate, and thousand-grain weight, and ultimately leading to yield loss (Wang et al., 2015a; Wu et al., 2021).

High temperature at the PI stage can lead to changes in various physiological processes closely associated with grain yield formation in rice. It can inhibit the differentiation of branches and spikelets by altering endogenous phytohormones and limiting the supply of assimilates (Cao et al., 2016; Wu et al., 2016, 2017), resulting in decreases in the number of spikelets (Soda et al., 2018) and grain filling rate and grain weight (Wu et al., 2023). Furthermore, high temperature at the PI stage can lead to higher pollen abnormality, reduced anther dehiscence, and lower pollen dispersal in heat-sensitive rice varieties (Hu et al., 2021, 2024). Under high temperature stress, premature programmed cell death triggered by elevated levels of abscisic acid (ABA) in anthers and the accumulation of reactive oxygen species (ROS) cause abnormal degradation of the tapetum (Zhao et al., 2023). Additionally, high temperature stress suppresses the activity of cell-wall invertase and vacuolar invertase, reducing the conversion of sucrose into glucose and fructose, thereby decreasing the accumulation of starch in anther (Guan et al., 2023). These effects will lead to pollen and spikelet sterility, particularly in heat-sensitive rice varieties. In recent years, the earlier onset of extreme heat events has increased the probability of high temperature at the PI stage of mid-season rice in the Yangtze River Basin (Dou et al., 2020). Therefore, it is essential to explore some effective measures to enhance the heat tolerance of rice at the PI stage to ensure rice yield and food security under climate change.

Stress priming refers to exposing the plants to a specific type of stress during early development, which can enhance the tolerance of plants to stresses that occur later, and is an effective strategy to improve plant tolerance to abiotic stresses. Exposure of plants to sub-lethal stress can enable the plants to “memorize” this stress and mount a stronger defense in subsequent stress events (Ganie et al., 2024). For instance, short-term high-temperature priming (at 37°C for 2 hours) at the seedling stage has been shown to increase cytokinin (CTK) levels via upregulating the expression of CTK synthesis genes in roots, and elevate jasmonic acid and auxin (IAA) levels in leaves, thus mitigating the damage caused by subsequent heat stress (Prerostova et al., 2023). However, the control of temperature is a huge challenge under field natural conditions, making it even impossible to adopt short-term high-temperature priming during early growth to improve heat tolerance during late growth.

Plants exhibit cross-tolerance to abiotic stresses, and prior exposure to sub-lethal stress can enhance their tolerance to subsequent abiotic stresses (De Pascali et al., 2022; Rossatto et al., 2023). Previous studies have demonstrated that drought priming during the vegetative phase enhances plant heat tolerance during the reproductive phase in wheat and rice by boosting antioxidant defenses, regulating hormone levels, and improving carbohydrate metabolism (Bahuguna et al., 2018; Kumar et al., 2023; Wang et al., 2015b; Zhao et al., 2024). However, rice is highly sensitive to soil water potential, and drought priming during early growth may reduce the yield if no high-temperature events occur later in the season (Bahuguna et al., 2018). Alternate wetting and drying irrigation (AWD) is a periodic water management practice that not only offers mild drought priming but also provides multiple physiological benefits. AWD boosts use efficiencies of water and nitrogen by increasing root activity and activities of key enzymes for nitrogen assimilation in rice (Gao et al., 2024; Li et al., 2022; Qi et al., 2024; Zhang et al., 2021). Secondly, AWD improves the photosynthetic activity (Zhang et al., 2021), promotes enzyme activities for synthesis and transformation of sucrose and starch in rice stems (Zang et al., 2024), companying more stem non-structural carbohydrates distributed to filling grains (Zang et al., 2024). Furthermore, AWD enhances the contents of cytokinins in panicles, roots, and root bleeding sap (Meng et al., 2025), and elevates the contents of IAA with a decreased ABA content in rice grains (Chen et al., 2025). Collectively, these physiological and biochemical modifications under AWD improve yield formation. Therefore, we hypothesize that AWD during early growth is an effective and low-risk strategy to enhance heat tolerance at the PI stage in rice. Based on this hypothesis, this study aims to examine the effects of AWD at the tillering stage on high-temperature tolerance at the PI stage. The findings are expected to facilitate the development of climate-resilient rice production systems for global food security under global climate change.

## 2 Materials and methods

### 2.1 Plant materials, growth conditions, and experimental design

The pot experiment was conducted from June to October 2019 at the experimental station of Huazhong Agricultural University, Wuhan, China (30°29′N, 114°22′E). Based on previous studies (Hu et al., 2024; Wu et al., 2019), four varieties were selected in terms of responses of panicle differentiation, spikelet fertility, grain yield formation to high temperatures, including Liangyoupeijiu (LYPJ, a two-line hybrid highly susceptible to heat), IR64 (an inbred variety highly susceptible to heat), Huanghuazhan (HHZ, an inbred variety moderately tolerant to heat) and Shanyou 63 (SY63, a three-line hybrid tolerant to heat stress).

Seeds were treated to break dormancy at 50°C for 2 days, sterilized by soaking in 10% H_2_O_2_ for 30 min, and thoroughly rinsed with water. The seeds were then soaked for 48 h for pre-germination, followed by sowing in plastic seedling trays with paddy loam soil and cultivating outdoors under moist conditions. Three uniform seedlings were transplanted into plastic pot (25.5 cm height × 24.4 cm diameter) at the three-leaf stage. The soil used was clay loam soil with pH of 6.6, containing 1.0 g kg^−1^ of total nitrogen, 8.1 mg kg^−1^ of available phosphorus, and 117.9 mg kg^−1^ of available potassium and 12.3 g kg^−1^ of organic matter.

The experiment was set up as a split-plot design, with temperature treatments assigned to main plots and irrigation regimes to the sub-plot. Two irrigation regimes were applied during the tillering stage: continuous flooding irrigation (CF) and AWD. The CF regime maintained a water depth of 2–3 cm in the pots until one week before harvest. The AWD regime was applied from 15 d after transplanting (seedlings were recovered from transplanting injury, Chu et al., 2014) to the 2^nd^ stage of PI stage (Hu et al., 2021). One tensiometer was installed in each plot at a soil depth of 15–20 cm, and readings were recorded daily at 8:00, 13:00, and 18:00. When the soil water potential dropped to −20 kPa, irrigation with a water depth of 2–3 cm was applied to the pots. According to Zhang et al. (2010) and Yang et al. (2017), the soil water potential of -25 – -15 kPa has no obvious effects on grain yield when AWD is performed. Seven times for AWD were implemented during the irrigation treatment period. The soil water potential generally declined from 0 kPa to –20 kPa within 1 – 3 d, depending on ambient temperature and irradiation conditions. A rain shelter was built at the experiment site and closed during rainfall events.

For temperature treatment, the plants were transferred to glass greenhouses (4 m length, 4 m width, and 4.5m height). Each greenhouse was equipped with air conditioners, dehumidifiers, circulation fans and LED floodlights. All the facilities were connected to a central programmable auto-control system (RHQH-4.3K-1X-T, Wuhan Ruihua Instrument & Equipment Co., Ltd, China). Two temperature treatments were started at the 2^nd^ stage of panicle development (Hu et al., 2021). The high daytime temperature treatment (HDT) simulated the actual increase in daytime temperature from 7:00 to 19:00 (the temperature was artificially set as 30°C from 7:00 to 9:00, 33°C from 9:00 to 10:00, 35°C from 10:00 to 11:00, 37°C from 11:00 to 15:00, 35°C from 15:00 to 16:00, 33°C from 16:00 to 17:00, and 30°C from 17:00 to 19:00), and the control treatment (CK) maintained a favorable temperature for rice plant growth during the entire day (the temperature was artificially set as 27°C from 7:00 to 9:00, 29°C from 9:00 to 10:00, 31°C from 10:00 to 11:00, 32°C from 11:00 to 15:00, 31°C from 15:00 to 16:00, 29°C from 16:00 to 17:00, and 27°C from 17:00 to 19:00) (**Supplementary Table S1**). The relative humidity (%) in the greenhouses was set at 75%. The nighttime temperature and relative humidity were set as 26°C and 75% from 19:00 to 7:00. Air temperature and relative humidity at 5 cm above the rice canopy were recorded by using HOBO (H08-003-02, Onset Computer Corporation, Bourne, MA, USA) with four sensors. The temperature treatments were carried out for successive 15 d. The actual mean daily maximum and minimum temperatures were 38.4°C and 26.9°C under HDT, and 32.4°C and 24.7°C under CK during the 15 d. The actual mean daily maximum and minimum relative humidity were 89.6% and 63.7% under HDT, and 82.4% and 60.0% under CK (**Supplementary Figure S1**).

Each treatment had three replicates, with each replicate consisting of seven pots. Each pot was manually rotated 90°clockwise every seven days to mitigate the positional effect, and the location of each replicate in the greenhouse was manually changed every five days. After termination of temperature treatment, plants were grown under natural light and temperature conditions until maturity. All pots maintained a water depth of 2–3 cm in the pots until one week before harvest. Pests, diseases, birds, and weeds were carefully managed to minimize their impact on the experimental results.

### 2.2 Estimation of grain yield and yield components

At maturity, three plants from three pots for each experimental replicate were harvested. After recording the number of effective panicles with more than five filled grains, all plants were divided into stems, leaves, and panicles. The harvested grains were oven-dried at 80°C to a constant weight for determination of grain yield (g per plant) and thousand-grain weight (g). Then, the filled and unfilled grains were divided by submersing in tap water, and empty grains were separated from the partially-filled grains by winnowing. After winnowing, all empty grains were pressed with a thumb to distinguish the partially filled grains from empty ones. The numbers of the three type grains were then counted. The seed setting rate was calculated as the number of filled grains divided by the total number of grains per plant. Spikelet fertility (%) was calculated as the number of filled and partially filled grains divided by the total number of grains per plant.

### 2.3 Measurement of pollen fertility

**Figure 1:**
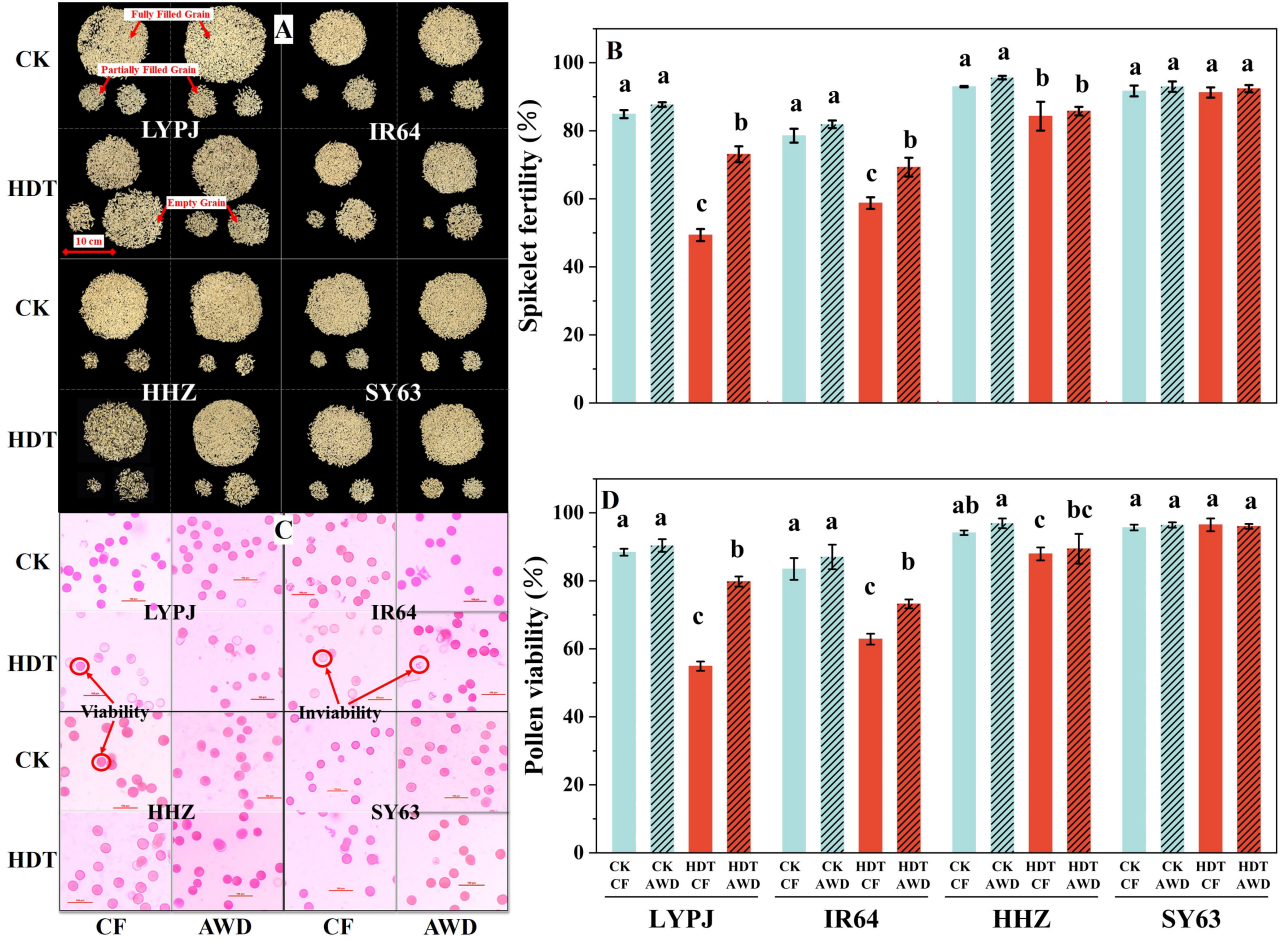
Effects of alternate wetting and drying irrigation at the tillering stage on spikelet fertility **(A**, **B)** and pollen viability **(C**, **D)** under high daytime temperature at the panicle initiation stage. Data are presented as mean ± standard deviation (n = 3). Different letters on the top of columns indicate statistical significant difference among the four treatment combinations across temperature and irrigation treatments for each variety at the *P* < 0.05 level by the least significant difference test. A: grains of LYPJ, IR64, HHZ and SY63 under the four combinations across temperature and irrigation treatments, respectively. The upper part on each figure represents fully filled grains, the lower left part represents partially filled grains, and the lower right part represents empty grains. C: pollen grains of LYPJ, IR64, HHZ and SY63 under the four combinations across temperature and irrigation treatments, respectively. The purplish-red and spherical pollen grains were considered as viable pollen grains, the unstained or deformed grains represented inviable pollen grains. CK, control temperature treatment; HDT, high daytime temperature treatment; CF, continuous flooding; AWD, alternate wetting and drying irrigation.

At heading, 15 unopened spikelets were pooled from the middle part of the three primary panicles that were about to emerge from three pots in each replicate. Anthers were carefully removed using forceps and placed on glass slides containing 60 μL of Alexander solution (Bioshap, China). The anthers were crushed to release the pollen grains, and the anther walls were discarded. After staining for 10 min, pollen grains were examined under an inverted fluorescence microscope (Ti-SR, Nikon, Tokyo, Japan), and five fields were observed for each replicate. Pollen grains that were stained purplish-red and had spherical shape were considered as viable pollen grains, while those that were unstained or deformed were considered as sterile pollen grains (**Figure 1**). The pollen viability (%) was calculated as the percentage of reddish-purple pollen grains to all pollen grains.

### 2.4 Measurement of panicle temperature, leaf area and photosynthetic rate, transpiration rate, and evapotranspiration

**Figure 2:**
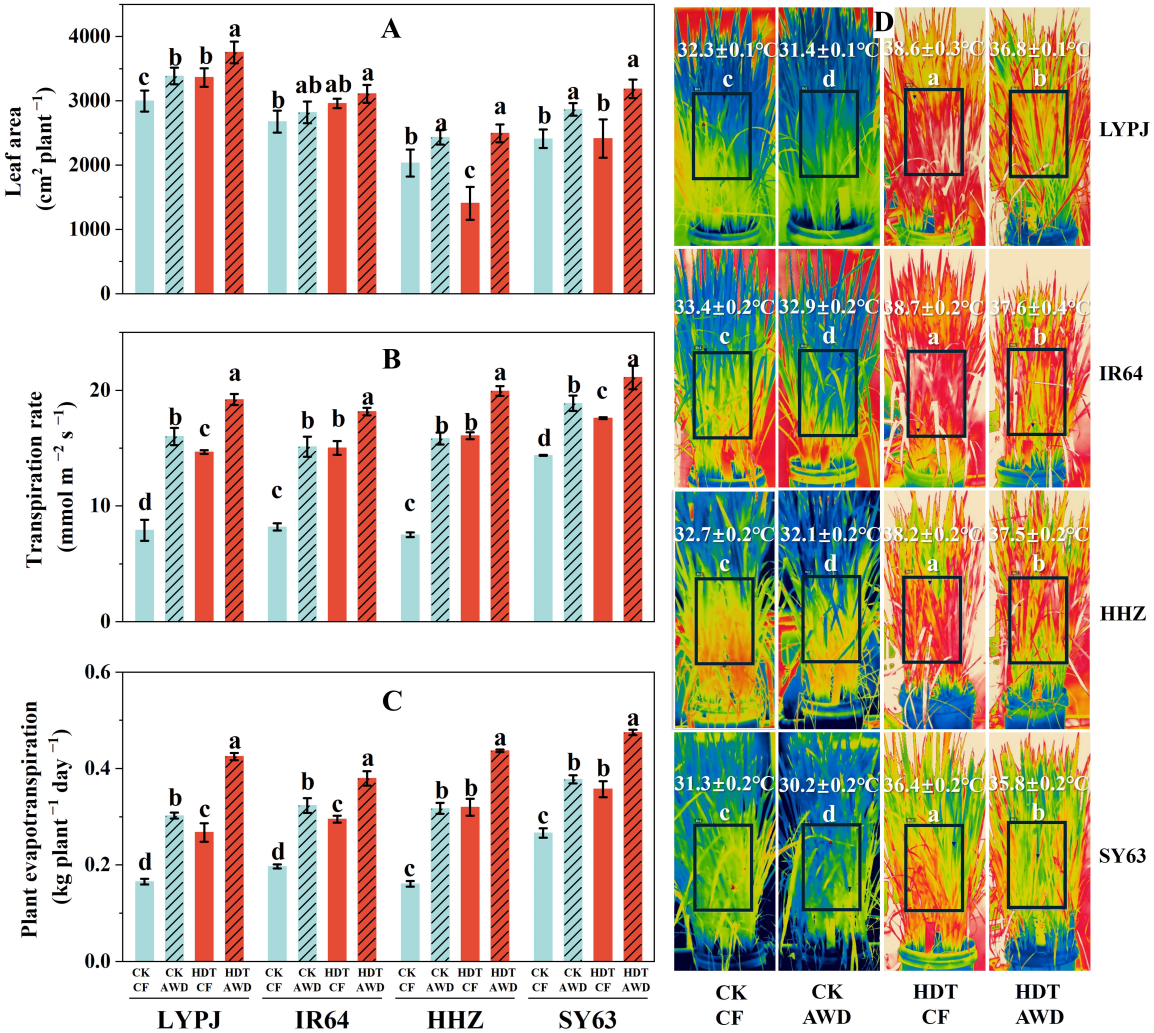
Effects of alternate wetting and drying irrigation at the tillering stage on leaf area **(A)**, transpiration rate **(B)**, plant evapotranspiration **(C)** and panicle temperature **(D)** under high daytime temperature at the panicle initiation stage. Data are presented as mean ± standard deviation (n = 3). Different letters on the top of columns indicate statistical significant difference among the four treatment combinations across temperature and irrigation treatments for each variety at the *P* < 0.05 level by the least significant difference test. CK, control temperature treatment; HDT, high daytime temperature treatment; CF, continuous flooding; AWD, alternate wetting and drying irrigation.

On the 10^th^ day after HDT, infrared thermographic images of aboveground plants were captured using a FLIR ThermaCAMTMS65 camera. Using ThermaCAM Researcher Pro 2.7 software (FLIR Systems Inc., Portland, OR, USA), a rectangle was set on the center of the plant to cover the panicle region (equal to approximately 0.06 m^2^), as showed in **Figure 2**, and the average temperature of the panicle region was subsequently determined with three plants per replicate.

All leaves were removed from three plants for each replicate, and a LI-3100 leaf area meter (LI-COR) was used to measure the leaf area of a portion of the leaves (about 500 cm^2^), the measured leaves and the remaining leaves were then dried to constant weight, and weighed, respectively, and the leaf area per plant (cm² plan^-1^) was calculated.

A portable gas-exchange system (LI-6800) was used to measure leaf photosynthetic rate and single-leaf transpiration rate from 09:00 to 15:00 on four successive clear sunny days. The measurement conditions were as follows: a light intensity of 1500 µmol m^-2^ s^-1^, an air flow rate of 500 µmol s^-1^, a reference CO_2_ concentration of 380–390 µmol mol^-1^, a leaf temperature of 30°C under CK and 37°C under HDT. The leaf chamber temperature of the instrument was set according to the average temperatures in the CK and HDT greenhouses during the measurement period (from 09:00 to 15:00), respectively. Five leaves for each replicate were used to measure the traits.

Three pots with plants were separately weighed at 19:00 after watering to a designated water layer and again the next day at the same time for each replicate. Plant evapotranspiration rate (kg plan^-1^ day^-1^) was calculated based on the weight difference between these two measurements.

### 2.5 Determination of panicle-related traits

To investigate the panicle-related traits, nine main tillers were collected from three pots for each experimental replicate upon the emergence of 1/2 to 3/4 of the main stem panicle. Any vestiges or small protrusions remaining on the panicle were considered as branches or florets that had degraded during panicle development (Mu et al., 2005). The numbers of existing primary branches and secondary branches, retrograded primary branches (RPB) and secondary branches (RSB), existing florets on primary branches and on secondary branches, retrograded florets on primary branches (RFPB) and on secondary branches (RFSB) were recorded. Then, the numbers of differentiated primary branches (PB) and differentiated secondary branches (SB), differentiated florets on primary branches (FPB) and differentiated florets on secondary branches (FSB), and spikelets per panicle were calculated.

### 2.6 Determination of non-structural carbohydrates in rice plants

Three uniform plants from three pots were collected at the termination of HDT, and then divided into stems (culms and sheaths), leaves, and panicles. All samples were oven-dried at 80°C to a constant weight, and finely ground for determination of non-structural carbohydrates (NSCs) according to Li et al. (2017). Briefly, 0.1 g of the sample powder was placed in a 10 mL centrifuge tube containing 5 mL ethanol (80%). The tube was incubated in an 80°C water bath for 30 min and then centrifuged at 6000 rpm for 5 min, and the supernatant was transferred to a 25 mL volumetric flask. The extraction process was repeated three times. All three supernatants were pooled in a flask, followed by the addition of distilled water to 25 mL for determination of soluble sugar and sucrose. Three measurements were conducted for each ground sample.

After evaporation of the remaining ethanol, 2 mL of distilled water was added into the tube with the precipitate, and the precipitate was gelatinized in a 100°C water bath for 15 min. After cooling, 2 mL of HClO_4_ (9.2 mol L^−1^) was added and mixed, and 2 mL of distilled water was added. Then, centrifugation was performed at 6000 rpm for 5 min, and the supernatant was collected. Subsequently, 2 mL HClO_4_ (4.6 mol L^−1^) was added to the tube, vortexed, followed by the addition of 2 mL distilled water. After another centrifugation at 6000 rpm for 5 min, the supernatant was collected. The two supernatants were thoroughly combined for starch measurement.

Anthrone colorimetric assay was used to measure concentrations of soluble sugar and starch. Absorbance readings were obtained at 620 nm using a Microplate Reader (Spectramax i3x, Molecular devices, California, USA), and the concentration (mg g^-1^) was determined by comparison with a glucose standard curve. The starch concentration was the corresponding concentration multiplied by 0.9. The concentration of NSCs (mg g^-1^) was determined by summing the soluble sugar and starch concentrations.

To measure the sucrose concentration, 0.1 mL of the soluble sugar extract was combined with 0.05 mL of NaOH solution (1 mol L^−1^) and incubated in a 100°C water bath for 5 min. Next, 0.2 mL of 0.1% resorcinol solution and 0.7 mL of HCl solution (9 mol L^−1^) were added. The mixture was reacted in an 80°C water bath for 10 min, and the absorbance was measured at 480 nm using a spectrophotometer. The sucrose concentration was calculated by comparison with a sucrose standard curve.

### 2.7 Quantitative analysis of sucrose metabolism-related enzyme activity

At the termination of HDT, three primary tillers were collected from three uniformly growing plants in each treatment. The tillers were divided into leaves, stems, panicles, and roots and thoroughly rinsed. All samples were flash-frozen in liquid nitrogen and stored at −80°C for subsequent enzyme activity in leaves, stems and panicles, phytohormone content and gene expression analyses in panicles and roots.

The activities of sucrose synthase in the cleavage direction (SSc, EC 2.4.1.13), and acid and neutral invertases (AI and NI, EC 3.2.1.26) were measured following the method of Li et al. (2017).

For extraction of SSc, AI, and NI, a total of 0.1 g of fresh panicles was homogenized in 2 mL of enzyme extraction buffer (150 mM Tris-HCl, pH 8.0; 10 mM MgCl_2_; 2 mM EDTA; 0.1 mM phenazine methosulfate; 1 mM benzamidine; 3% (w/v) polyvinylpolypyrrolidone; 10 mM ascorbic acid) at 4°C. The homogenate was centrifuged at 12,000 rpm and 4°C for 20 min, and the supernatant was collected for measurement of SSc, AI and NI. The enzyme extraction was repeated three times.

For SSc activity, 0.1 mL of enzyme extract was mixed with 0.3 mL of reaction buffer (100 mM Hepes-NaOH, pH 5.5; 5 mM NaCl; 1.8 mM UDP) and 0.1 mL of 100 mM sucrose. The mixture was incubated at 30°C for 30 min, and the reaction was terminated by heating at 100°C for 1 min. Next, 0.5 mL of 3,5-dinitrosalicylic acid reagent (28.5 mM) was added, followed by heating at 100°C for 5 min. After cooling, the absorbance was measured at 540 nm. The inactivated enzyme extract by heating at 100°C for 1 min was used as the control. For AI and NI activity, 0.1 mL of enzyme extract was combined with 0.3 mL of 100 mM sodium acetate buffer (pH 4.8) for AI, and 0.3 mL of 40 mM phosphate buffer (pH 7.0) for NI, and then 0.1 mL of 100 mM sucrose solution was added, respectively. The subsequent steps were identical to those for SSc. The inactivated enzyme extract by heating at 100°C for 1 min was used as the control. A glucose standard curve was used to determine the amount of produced glucose. SSc, AI, and NI activities were expressed as the amount of glucose produced per milligram of protein per hour (µmol glucose mg^-1^ protein h^-1^). Protein content in the enzyme extract was measured according to Bradford (1976) using bovine serum albumin as the standard. Three technical replicates were performed for each enzyme extraction.

### 2.8 Extraction and content determination of phytohormones

At the termination of HDT, three uniformly growing plants in each treatment were selected, and three primary tiller panicles and 20 newly-grown roots with a length of approximately 8 cm were collected for determination of phytohormones and RNA extraction.

A total of 0.1 g of fresh tissue was ground in liquid nitrogen and transferred to a centrifuge tube, followed by the addition of 1 mL of 75% methanol solution (methanol: ultrapure water: formic acid = 75: 20: 5), and then the sample was shaken at 4°C for 12 h. After centrifugation at 12,000 rpm and 4°C for 15 min, the supernatant was filtered through a 0.23 µm membrane into a 2 mL vial, the filtrate was collected for determination of hormone concentration, using the liquid chromatography–triple quadrupole mass spectrometer system (LC-30 AD/MS-8050 system, Shimadzu, Japan). An external standard method was employed to quantify 12 plant hormones in rice tissue, including *N* ^6^-(Δ^2^-isopentenyl)adenine(iP), isopentenyladenine riboside(iPR), isopentenyladenine riboside-5’-monophosphate(iPMP), isopentenyladenine 9-glucoside(iP9G), *trans-*zeatin(tZ), *trans-*zeatin riboside(tZR), *trans-*zeatin 9-glucoside(tZ9G), GA_1_, GA_3_, GA_4_, IAA, ABA. Phytohormone extraction was repeated three times for each biological replicate.

The flow rate was 0.4 mL min^-1^; the column temperature was 40°C; and the injection volume was 5 µL. Detailed gradient conditions for elution are listed in **Supplementary Table S2**. Hormone standards (OlChemlm Ltd., Czech Republic) were dissolved in pure HPLC-grade methanol and prepared at 13 concentrations (0.01, 0.02, 0.05, 0.1, 0.2, 0.5, 1, 2, 5, 10, 20, 50 ppb).

For recovery assessment, two samples (each 0.1 g of fresh tissue) from the same treatment were separately ground, and the above-mentioned procedure was performed for extraction of plant hormones. One of the two filtrates was mixed with 0.5 mL of 1 ppb standard hormone samples containing 12 hormones, and then the hormone contents in the two filtrates were determined. According to the concentration difference of the two filtrates, recovery was calculated, which was 89.7 ± 3.7%, 97.1 ± 5.2%, 91 ± 6%, 91.8 ± 4.3%, 95.9 ± 1.8%, 93.7 ± 1.7%, 103 ± 3.6%, 105 ± 7.8%, 98.6 ± 7%, 102.8 ± 1.3%, 93.7 ± 8.5%, and 82.3 ± 2.2% for iP, iPR, iPMP, iP9G, tZ, tZR, tZ9G, GA_1_, GA_2_, GA_3_, IAA, and ABA, respectively. In this study, the bound CTKs included tZ9G and iP9G as inactive CTKs (iCTKs), and other CTKs (tZ, tZR, iP, iPR, and iPMP) were designated as active CTKs (aCTKs).

### 2.9 RNA extraction and real-time RT-qPCR analysis of CTK metabolism-related genes

Total RNA was extracted, reverse-transcribed, and subjected to quantitative real-time PCR (qRT-PCR) following the procedure described in a previous study (Liu et al., 2023). Total RNA was extracted from plant tissues using TRNzol Universal Reagent (TIANGEN, DP424) following the manufacturer’s protocol. Briefly, 0.1 g of fresh plant material was ground in liquid nitrogen, then mixed with 1 mL of TRNzol and vortexed at room temperature for 5 min. Subsequently, 0.2 mL of chloroform was added and the mixture was inverted for 2 min, and then incubated at room temperature for 3 min. After centrifugation at 12,000 rpm at 4°C for 15 min, 0.5 mL of the supernatant was transferred to another tube. An equal volume (0.5 mL) of isopropanol was added, mixed thoroughly, and incubated at room temperature for 10 min. The mixture was then centrifuged again (12,000 rpm, 4°C, 15 minutes), and the supernatant was discarded. The pellet was washed once with 1 mL of 75% ethanol, centrifuged at 12,000 rpm and 4°C for 10 min, carefully dried at room temperature, and resuspended in 20 µL of ddH_2_O to dissolve the RNA.

RNA concentration and purity were measured on a TGem Plus full-wavelength spectrophotometer (TIANGEN, OSE-260-01/02). Genomic DNA was removed using DNase I (Invitrogen, USA), and first-strand cDNA was synthesized from DNase-treated RNA with M-MLV reverse transcriptase (Invitrogen, USA) according to the manufacturer’s protocol. The resulting cDNA was then ten-fold diluted for qRT-PCR. qRT-PCR was conducted on an ABI ViiA™ 7 Real-Time PCR System (Applied Biosystems, USA) using the Roche FastStart Universal SYBR Green Master (Rox) Kit (Roche, Switzerland). The cycling program included an initial denaturation at 95°C for 2 min, followed by 45 cycles of 95°C for 10 s, 60°C for 10 s, and 72°C for 20 s. The housekeeping rice gene *Actin* was used as the internal control and cytokinin synthesis- and degradation-related genes were quantified with gene-specific primers listed in **Supplementary Table S3**. Relative gene expression was determined using the 2^-ΔΔCT^ method (Livak and Schmittgen, 2001).

The expression level of panicle *LOG* gene and root *LOG2* gene in LYPJ under CF-CK was set as 1, and the expression of other genes in panicles and roots was calculated with the expression of these two genes as reference, respectively. Quantification of RNA expression was repeated three times for each biological replicate.

### 2.10 Statistical analysis

The value for a given investigated trait was presented as the mean of three replicates with standard deviation. Statistical significance was assessed at the 5% probability level with the least significant difference test using Statistix 9 (Analytical Software, Tallahassee, FL, USA), comparing four treatment combinations across irrigation regime and temperature treatment for each variety. The principal component analysis (PCA) was performed with Origin 24.0 using physiological traits significantly correlated with yield, spikelet number, spikelet fertility and pollen viability across the four varieties and two irrigation treatments under HDT, respectively (**Supplementary Table S4**). Pearson correlation analysis was performed. Graphs were generated with Origin 24.0, and the standard deviation of the mean was calculated and represented in the graphs as error bars.

## 3 Results

### 3.1 Effects of alternate wetting and drying irrigation on yield and yield components

Under CF, HDT significantly decreased the grain yield of LYPJ, IR64, and HHZ by 63%, 55%, and 32%, respectively (**Table 1**), and only led to an insignificant reduction in grain yield of SY63. HDT showed consistent effects on the grain yield of four varieties under AWD and CF. Under CK, AWD significantly increased the grain yield of LYPJ (28%), IR64 (18%), HHZ (19%), and SY63 (19%). Similar increases were obtained under HDT, and the increase degree of yield induced by AWD was greater compared to that under CK conditions.

**Table 1:** Effects of alternate wetting and drying irrigation at the tillering stage on yield and yield components under high daytime temperature at the panicle initiation stage.

Variety	Temperature	Irrigation	Yield per plant	Panicles per plant	Grains per panicle	Thousand grain weight	Seed setting rate
			g plant^−1^	No. plant^−1^	No. panicle^−1^	g	%
LYPJ	CK	CF	25.7 ± 0.9 b	9.8 ± 0.7 a	171.3 ± 1.2 b	20.9 ± 0.6 a	73.4 ± 1.4 a
		AWD	33.0 ± 1.1 a	10.6 ± 0.4 a	191.9 ± 3.5 a	21.1 ± 0.8 a	77.6 ± 3.1 a
	HDT	CF	9.5 ± 2.4 d	9.3 ± 1.7 a	142.3 ± 9.5 c	18.5 ± 1.0 b	39.2 ± 3.7 c
		AWD	20.4 ± 1.0 c	9.9 ± 0.2 a	169.5 ± 6.1 b	20.1 ± 0.1 a	60.7 ± 0.8 b
IR64	CK	CF	18.7 ± 1.2 b	14.3 ± 0.6 a	81.6 ± 3.1 b	21.7 ± 0.4 a	73.6 ± 1.7 b
		AWD	22.1 ± 0.4 a	14.2 ± 0.2 a	89.4 ± 0.7 a	22.2 ± 0.8 a	78.6 ± 0.8 a
	HDT	CF	8.4 ± 0.3 d	14.8 ± 1.1 a	58.1 ± 3.5 d	19.2 ± 0.3 b	51.0 ± 1.1 d
		AWD	13.5 ± 1.7 c	14.4 ± 1.0 a	68.4 ± 1.0 c	20.2 ± 0.9 b	66.8 ± 2.6 c
HHZ	CK	CF	32.0 ± 1.2 b	13.4 ± 0.2 a	126.0 ± 1.1 b	21.0 ± 0.5 a	90.2 ± 0.5 a
		AWD	38.2 ± 0.7 a	13.7 ± 0.3 a	143.5 ± 1.1 a	21.0 ± 0.2 a	93.1 ± 1.2 a
	HDT	CF	21.7 ± 0.2 d	12.9 ± 0.8 a	115.5 ± 2.8 c	18.0 ± 0.4 b	80.8 ± 3.7 b
		AWD	24.2 ± 2.0 c	13.1 ± 0.5 a	123.2 ± 2.6 b	18.0 ± 0.4 b	82.1 ± 2.5 b
SY63	CK	CF	29.4 ± 2.8 b	12.4 ± 1.1 a	107.0 ± 2.8 b	25.6 ± 0.5 a	86.7 ± 5.0 a
		AWD	35.1 ± 2.9 a	12.4 ± 0.5 a	126.4 ± 9.3 a	25.7 ± 0.3 a	86.9 ± 0.2 a
	HDT	CF	27.9 ± 1.3 b	12.4 ± 0.2 a	103.1 ± 3.0 b	25.2 ± 0.7 a	86.5 ± 1.6 a
		AWD	32.7 ± 2.3 ab	12.0 ± 1.2 a	125.5 ± 2.2 a	25.7 ± 0.5 a	84.8 ± 0.2 a

Data are presented as mean ± standard deviation (n = 3). Different letters within an identical column indicate statistical significant difference among the four treatment combinations across temperature and irrigation treatments for each variety at the *P* < 0.05 level by the least significant difference test. CK, control temperature treatment; HDT, high daytime temperature treatment; CF, continuous flooding; AWD, alternate wetting and drying irrigation.

HDT significantly reduced the number of grains per panicle and seed setting rate of LYPJ, IR64, and HHZ under both CF and AWD, but showed no significant effect on these traits of SY63. Under AWD, HDT significantly reduced the grain weight of IR64 and HHZ. Under CK, AWD significantly increased the number of grains per panicle in all four varieties, but had no significant effect on the grain weight and seed setting rate except for IR64. Under HDT, AWD significantly increased the number of grains per panicle of all four varieties, elevated the grain weight and seed setting rate of LYPJ and IR64, while showing no significant effect on grain weight and seed setting rate of HHZ and SY63 (**Table 1**).

### 3.2 Effects of alternate wetting and drying irrigation on spikelet fertility and pollen viability

Under CF, HDT significantly reduced the spikelet fertility of LYPJ, IR64, and HHZ by 42%, 25%, and 9%, respectively (**Figure 1B**), which could be primarily attributed to a decrease in filled grains and a substantial increase in empty grains (**Figure 1A**), but had no significant effect on the spikelet fertility of SY63. The effect of HDT on spikelet fertility of four varieties under AWD was consistent with that under CF. AWD showed no significant effect on the spikelet fertility of four varieties under CK. Under HDT, AWD significantly increased the spikelet fertility by 48% in LYPJ and 17% in IR64, while had no obvious effect on the spikelet fertility of HHZ and SY63 (**Figure 1B**).

Under CK, the CF and AWD treatments led to large numbers of purplish-red, spherical pollen grains while few light pink, deformed pollen grains in all four rice varieties (**Figure 1C**). In contrast, HDT significantly increased the number of inviable pollen grains (unstained and deformed) and therefore decreased the pollen viability of LYPJ, IR64, and HHZ under both CF and AWD (**Figures 1C**, **D**). Under CK, AWD showed no significant effect on the pollen viability of four varieties. Under HDT, AWD significantly enhanced the pollen viability of LYPJ and IR64 compared with CF (**Figure 1D**).

### 3.3 Effects of alternate wetting and drying irrigation on panicle-related traits

Under CF, HDT significantly decreased the spikelet number of LYPJ (21%), IR64 (12%), and HHZ (8%), while had no significant effect on that of SY63 (**Table 2**). The effect of HDT on the spikelet number of four varieties was consistent under AWD and CF. AWD increased the spikelet number of all varieties under both CK and HDT.

**Table 2:** Effects of alternate wetting and drying irrigation at the tillering stage on panicle-related traits under high daytime temperature at the panicle initiation stage.

Variety	Temperature	Irrigation	PB	RPB	SB	RSB	FPB	RFPB	FSB	RFSB	Spikelets
			No. panicle^−1^	No. panicle^−1^	No. panicle^−1^	No. panicle^−1^	No. panicle^−1^	No. panicle^−1^	No. panicle^−1^	No. panicle^−1^	No. panicle^−1^
LYPJ	CK	CF	12.4 ± 0.2 ab	0.1 ± 0.1 ab	58.8 ± 1.3 a	21.3 ± 1.4 b	74.1 ± 1.5 a	0.1 ± 0.2 a	112.4 ± 4.6 b	4.5 ± 0.3 c	181.9 ± 5.6 b
		AWD	12.3 ± 0.1 ab	0.0 ± 0.1 ab	59.7 ± 0.9 a	15.5 ± 0.7 c	75.2 ± 0.4 a	0.0 ± 0.0 a	132.3 ± 0.3 a	1.3 ± 0.2 d	206.3 ± 0.6 a
	HDT	CF	11.9 ± 0.5 b	0.3 ± 0.3 a	54.9 ± 2.8 b	25.2 ± 2.2 a	64.2 ± 2.6 b	0.3 ± 0.3 a	98.7 ± 1.8 c	18.2 ± 1.5 a	144.4 ± 2.3 c
		AWD	12.6 ± 0.2 a	0.0 ± 0.0 b	60.3 ± 1.5 a	21.2 ± 1.1 b	75.2 ± 1.0 a	0.0 ± 0.0 a	113.7 ± 1.8 b	8.4 ± 0.1 b	180.5 ± 1.1 b
IR64	CK	CF	10.0 ± 0.2 a	0.0 ± 0.0 a	24.4 ± 0.4 ab	10.0 ± 0.4 b	53.0 ± 1.3 ab	0.1 ± 0.1 b	49.5 ± 0.1 b	7.6 ± 1.0 c	94.8 ± 1.0 b
		AWD	10.0 ± 0.2 a	0.0 ± 0.0 a	25.6 ± 0.2 a	8.4 ± 0.1 c	54.0 ± 0.8 a	0.0 ± 0.0 b	54.9 ± 1.1 a	4.8 ± 0.6 d	104.1 ± 2.1 a
	HDT	CF	10.0 ± 0.2 a	0.1 ± 0.2 a	24.0 ± 0.3 b	11.9 ± 0.3 a	51.4 ± 1.5 b	0.4 ± 0.2 a	44.5 ± 1.1 c	15.1 ± 0.3 a	80.4 ± 1.8 d
		AWD	10.0 ± 0.1 a	0.0 ± 0.0 a	25.1 ± 1.3 ab	11.6 ± 1.0 a	53.3 ± 0.8 ab	0.0 ± 0.0 b	48.0 ± 1.2 b	11.1 ± 0.7 b	90.1 ± 1.5 c
HHZ	CK	CF	13.0 ± 0.1 ab	0.0 ± 0.1 a	46.8 ± 0.9 a	15.0 ± 1.2 ab	74.5 ± 0.7 a	0.4 ± 0.3 a	94.5 ± 2.1 b	5.8 ± 0.2 b	159.9 ± 4.4 c
		AWD	13.2 ± 0.2 a	0.0 ± 0.1 a	46.1 ± 1.5 a	10.3 ± 0.5 c	74.0 ± 0.6 a	0.1 ± 0.1 a	105.7 ± 3.0 a	4.3 ± 0.4 c	175.3 ± 2.7 a
	HDT	CF	12.7 ± 0.1 b	0.3 ± 0.2 a	43.4 ± 0.2 b	16.3 ± 0.2 a	69.9 ± 2.1 b	0.6 ± 0.2 a	87.4 ± 1.6 c	10.1 ± 0.6 a	146.6 ± 4.0 d
		AWD	13.1 ± 0.2 a	0.1 ± 0.1 a	46.8 ± 1.5 a	13.9 ± 0.4 b	75.0 ± 1.1 a	0.2 ± 0.2 a	98.2 ± 3.0 b	5.7 ± 0.7 b	167.4 ± 3.4 b
SY63	CK	CF	11.2 ± 0.5 a	0.0 ± 0.0 a	41.4 ± 1.3 ab	10.4 ± 0.2 a	67.3 ± 3.3 a	0.2 ± 0.2 a	94.4 ± 4.1 b	4.2 ± 0.7 b	157.3 ± 6.4 b
		AWD	11.7 ± 0.6 a	0.0 ± 0.1 a	42.5 ± 1.2 ab	9.0 ± 1.2 a	70.2 ± 3.1 a	0.0 ± 0.1 a	102.3 ± 1.0 a	1.6 ± 0.6 c	170.9 ± 2.7 a
	HDT	CF	11.1 ± 0.4 a	0.1 ± 0.1 a	40.4 ± 1.4 b	10.3 ± 0.9 a	66.9 ± 2.0 a	0.0 ± 0.1 a	93.5 ± 1.5 b	6.0 ± 0.5 a	154.4 ± 2.4 b
		AWD	11.6 ± 0.5 a	0.1 ± 0.1 a	43.1 ± 1.1 a	9.7 ± 0.3 a	69.7 ± 2.6 a	0.0 ± 0.0 a	103.1 ± 2.2 a	3.4 ± 0.6 b	169.4 ± 2.4 a

Data are presented as mean ± standard deviation (n = 3). Different letters within an identical column indicate statistical significant difference among the four combinations across temperature and irrigation treatments for each variety at the *P* < 0.05 level by the least significant difference test. CK, control temperature treatment; HDT, high daytime temperature treatment; CF, continuous flooding; AWD, alternate wetting and drying irrigation. PB, differentiated primary branches; RPB, retrograded primary branches; SB, differentiated secondary branches; RSB, retrograded secondary branches; FPB, differentiated florets on primary branches; RFPB, retrograded florets on primary branches; FSB, differentiated florets on secondary branches; RFSB, retrograded florets on secondary branches.

Compared with CK, HDT showed no significant effect on the number of PB in four varieties under both CF and AWD, but significantly reduced the number of SB in LYPJ and HHZ under CF. Additionally, compared with CK, HDT significantly increased the number of RSB in LYPJ and IR64 under CF, and in LYPJ, IR64, and HHZ under AWD. AWD had no significant effect on the number of PB and SB in four varieties under CK, but significantly increased the number of PB in LYPJ and HHZ and that of SB in LYPJ, HHZ, and SY63 under HDT. Moreover, AWD significantly reduced the number of RSB in LYPJ, IR64 and HHZ under CK, and in LYPJ and HHZ under HDT (**Table 2**).

Under CF, HDT significantly reduced the number of FPB in LYPJ and HHZ, and the number of FSB in LYPJ, IR64, and HHZ. Under AWD, HDT showed no significant effect on the number of FPB in four varieties, but significantly reduced that of FSB in LYPJ, IR64, and HHZ. In addition, HDT significantly increased the number of RFSB in all four varieties under both CF and AWD. Under CK, AWD had no significant effect on the number of FPB in four varieties, while significantly increased that of FSB in all varieties. AWD significantly increased the number of FPB in LYPJ and HHZ and that of FSB in four varieties under HDT, but significantly reduced the number of RFSB in four varieties under both CK and HDT (**Table 2**).

### 3.4 Effects of alternate wetting and drying irrigation on leaf area, transpiration rate, plant evapotranspiration and temperature of panicle region

Under CF, HDT significantly increased the leaf area per plant of LYPJ, and had no significant effect on IR64 and SY63, while decreased the leaf area per plant of HHZ (**Figure 2A**). Under AWD, HDT also notably increased the leaf area of LYPJ, while had no significant effect on that of IR64, HHZ, and SY63. Compared with CF, AWD obviously increased the leaf area of LYPJ, HHZ, and SY63 under both CK and HDT, but had no evident effect on that of IR64.

In addition, HDT significantly enhanced the single-leaf transpiration rate and plant evapotranspiration of all four varieties under both CF and AWD, particularly under CF (**Figures 2B, C**). Similar results were obtained for AWD under both CK and HDT, and the increase was more pronounced under CK.

HDT resulted in a higher average temperature of panicle region than CK for all varieties under both CF and AWD (**Figure 2D**). Moreover, AWD led to a lower temperature than CF for all varieties under both CK and HDT. Specifically, compared with CF, AWD reduced the temperature of LYPJ, IR64, HHZ, and SY63 by 0.9°C, 0.5°C, 0.6°C, and 1.1°C under CK, and by 1.8°C, 1.1°C, 0.7°C, and 0.6°C under HDT, respectively. Moreover, SY63 exhibited the lowest average temperature across all treatments among the four varieties. Compared with that of LYPJ, the average temperature of panicle region of SY63 decreased by 1.0°C, 1.2°C, 2.2°C and 1.0°C under CK-CF, CK-AWD, HDT-CF, and HDT-AWD treatments, respectively.

### 3.5 Effects of alternate wetting and drying irrigation on dry matter and panicle non-structural carbohydrates

HDT significantly decreased the leaf net photosynthetic rate of LYPJ and IR64 under CF, while notably reduced the rate of IR64 under AWD (**Table 3**). AWD obviously elevated the photosynthetic rate of IR64 and SY63 under CK, while significantly increased the photosynthetic rate of LYPJ, IR64, and SY63 under HDT. HDT markedly reduced the panicle dry weight of LYPJ, IR64, and HHZ under both CF and AWD. AWD significantly increased the panicle dry weight of IR64, HHZ, and SY63 under CK, and enhanced that of all four varieties under HDT.

**Table 3:** Effects of alternate wetting and drying irrigation at the tillering stage on leaf photosynthetic rate, panicle dry matter and carbohydrate contents in panicles under high daytime temperature at the panicle initiation stage.

Variety	Temperature	Irrigation	Net photosynthetic rate	Panicle	Carbohydrate content in panicles			
				dry matter	NSCs	Soluble sugar	Starch	Sucrose
			µmol m^−2^ s^−1^	g plant^−1^	mg g ^−1^	mg g ^−1^	mg g ^−1^	mg g ^−1^
LYPJ	CK	CF	23.7 ± 0.7 a	1.4 ± 0.1 a	239.7 ± 3.9 b	179.7 ± 4.5 b	60.0 ± 3.2 b	127.8 ± 2.6 b
		AWD	25.0 ± 1.2 a	1.6 ± 0.2 a	267.0 ± 5.4 a	202.2 ± 3.2 a	64.8 ± 2.2 a	148.9 ± 0.7 a
	HDT	CF	21.6 ± 0.6 b	0.7 ± 0.0 c	170.9 ± 2.8 d	123.5 ± 1.8 d	47.4 ± 1.1 c	78.0 ± 1.5 d
		AWD	23.8 ± 0.8 a	1.0 ± 0.1 b	217.0 ± 5.5 c	159.2 ± 3.8 c	57.8 ± 1.7 b	115.3 ± 4.8 c
Correlation coefficient between pollen viability and carbohydrates content					0.95***	0.94***	0.92***	0.95***
IR64	CK	CF	19.2 ± 0.1 c	1.3 ± 0.1 b	236.5 ± 4.3 a	175.3 ± 4.9 a	61.2 ± 0.7 a	119.4 ± 4.6 a
		AWD	22.0 ± 0.5 a	1.5 ± 0.1 a	237.7 ± 5.0 a	178.7 ± 3.2 a	59.0 ± 1.9 a	124.9 ± 5.2 a
	HDT	CF	17.7 ± 0.2 d	0.9 ± 0.1 c	179.8 ± 2.9 c	128.9 ± 2.7 c	50.9 ± 1.2 b	84.0 ± 4.7 c
		AWD	20.7 ± 0.1 b	1.2 ± 0.0 b	192.5 ± 5.2 b	140.7 ± 4.5 b	51.8 ± 1.3 b	95.5 ± 3.0 b
Correlation coefficient between pollen viability and carbohydrates content					0.93***	0.94***	0.85***	0.98***
HHZ	CK	CF	22.6 ± 0.3 ab	0.5 ± 0.0 c	230.1 ± 2.4 b	173.9 ± 1.4 b	56.2 ± 1.0 b	121.8 ± 1.5 b
		AWD	23.9 ± 0.1 a	0.8 ± 0.0 a	248.6 ± 4.4 a	189.7 ± 3.7 a	58.8 ± 0.7 a	136.3 ± 3.8 a
	HDT	CF	22.2 ± 1.3 b	0.4 ± 0.0 d	195.0 ± 3.0 c	143.3 ± 1.6 c	51.7 ± 1.5 c	96.5 ± 4.8 c
		AWD	23.3 ± 0.5 ab	0.6 ± 0.0 b	229.2 ± 3.3 b	171.8 ± 2.8 b	57.4 ± 1.3 ab	119.4 ± 5.3 b
Correlation coefficient between pollen viability and carbohydrates content					0.74**	0.74**	0.67*	0.76**
SY63	CK	CF	22.2 ± 0.6 b	1.4 ± 0.2 b	281.4 ± 6.8 b	218.3 ± 4.9 b	63.1 ± 2.0 b	153.7 ± 2.7 b
		AWD	24.6 ± 0.4 a	1.9 ± 0.2 a	322.6 ± 7.6 a	249.7 ± 7.1 a	72.9 ± 1.3 a	178.5 ± 5.7 a
	HDT	CF	22.1 ± 0.2 b	1.4 ± 0.1 b	275.7 ± 4.4 b	212.9 ± 3.4 b	62.8 ± 1.0 b	149.8 ± 2.1 b
		AWD	24.3 ± 0.7 a	1.8 ± 0.0 a	313.5 ± 4.2 a	242.2 ± 1.2 a	71.3 ± 3.1 a	178.7 ± 1.1 a
Correlation coefficient between pollen viability and carbohydrates content					0.07^ns^	0.07^ns^	0.06^ns^	0.06^ns^

Data are presented as mean ± standard deviation (n = 3). Different letters within an identical column indicate statistical significant difference among the four treatment combination across temperature and irrigation treatments for each variety at the *P* < 0.05 level by the least significant difference test (LSD). CK, control temperature treatment; HDT, high daytime temperature treatment; CF, continuous flooding; AWD, alternate wetting and drying irrigation. ***, **, *: represents correlation significance at 0.001, 0.01, and 0.05 level, ns: regression not significance.

Furthermore, HDT significantly reduced the panicle NSCs content of LYPJ (29%), IR64 (24%), and HHZ (15%) under CF, while resulted in no significant decrease of panicle NSCs content in SY63 (**Table 3**). Specifically, HDT reduced the starch content of LYPJ, IR64, and HHZ by 21%, 17%, and 8%, soluble sugar content by 31%, 26%, and 18%, and sucrose content by 39%, 30%, and 21% under CF, respectively. The effect of HDT on panicle NSCs content of four varieties was consistent under AWD and CF. Under CK, AWD increased the panicle NSCs content of LYPJ, HHZ, and SY63 by 11%, 8%, and 15%, respectively; under HDT, AWD markedly increased the panicle NSCs content of LYPJ, IR64, HHZ, and SY63 by 27%, 7%, 18%, and 14%, respectively. In addition, AWD also increased the starch content, soluble sugar content, and sucrose content of LYPJ, HHZ, and SY63 under both HDT and CK, and enhanced the soluble sugar content and sucrose content of IR64 under HDT.

Additionally, the pollen viability of LYPJ, IR64, and HHZ was significantly positively correlated with the contents of total NSCs, starch, soluble sugar, and sucrose in panicles, particularly with the sucrose content (**Table 3**).

### 3.6 Effects of alternate wetting and drying irrigation on the activity of sucrose-metabolizing enzymes in panicles

**Figure 3:**
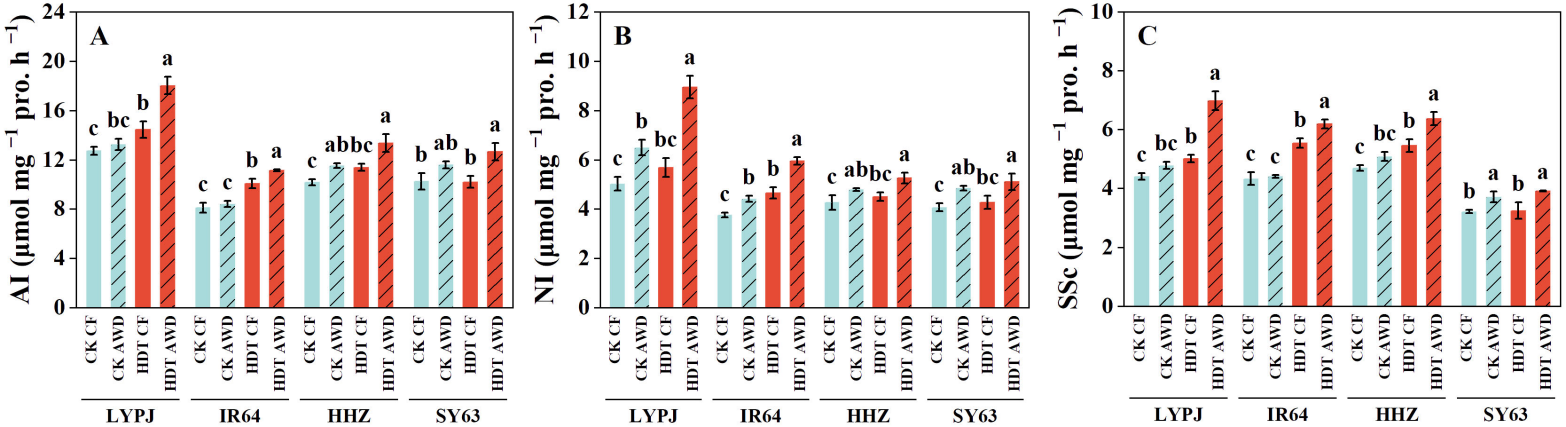
Effects of alternate wetting and drying irrigation at the tillering stage on enzyme activities [**(A)** for acid invertase; **(B)** for neutral invertase; **(C)** for sucrose synthase] for sucrose hydrolysis in panicles under high daytime temperature at the panicle initiation stage. Data are presented as mean ± standard deviation (n = 3). Different letters on the top of columns indicate statistical significant difference among the four treatment combinations across temperature and irrigation treatments for each variety at the *P* < 0.05 level by the least significant difference test. CK, control temperature treatment; HDT, high daytime temperature treatment; CF, continuous flooding; AWD, alternate wetting and drying irrigation; AI, acid invertase; NI, neutral invertase; SSc, Sucrose synthase in the cleavage direction.

Under CF, HDT increased the panicle AI activity in LYPJ and IR64, NI activity in IR64, and SSc activity in LYPJ, IR64, and HHZ (**Figure 3**). However, under AWD, HDT significantly increased the AI and NI activity in LYPJ and IR64 and SSc activity in LYPJ, IR64, and HHZ. HDT showed no obvious effect on the activities of AI, NI, and SSc in SY63. Under CK, AWD significantly increased the AI activity in HHZ, NI activity in all four varieties, and SSc activity in SY63. Under HDT, AWD significantly enhanced the activity of AI, NI, and SSc in all four varieties.

### 3.7 Effects of alternate wetting and drying irrigation on phytohormone concentration in panicles

**Figure 4:**
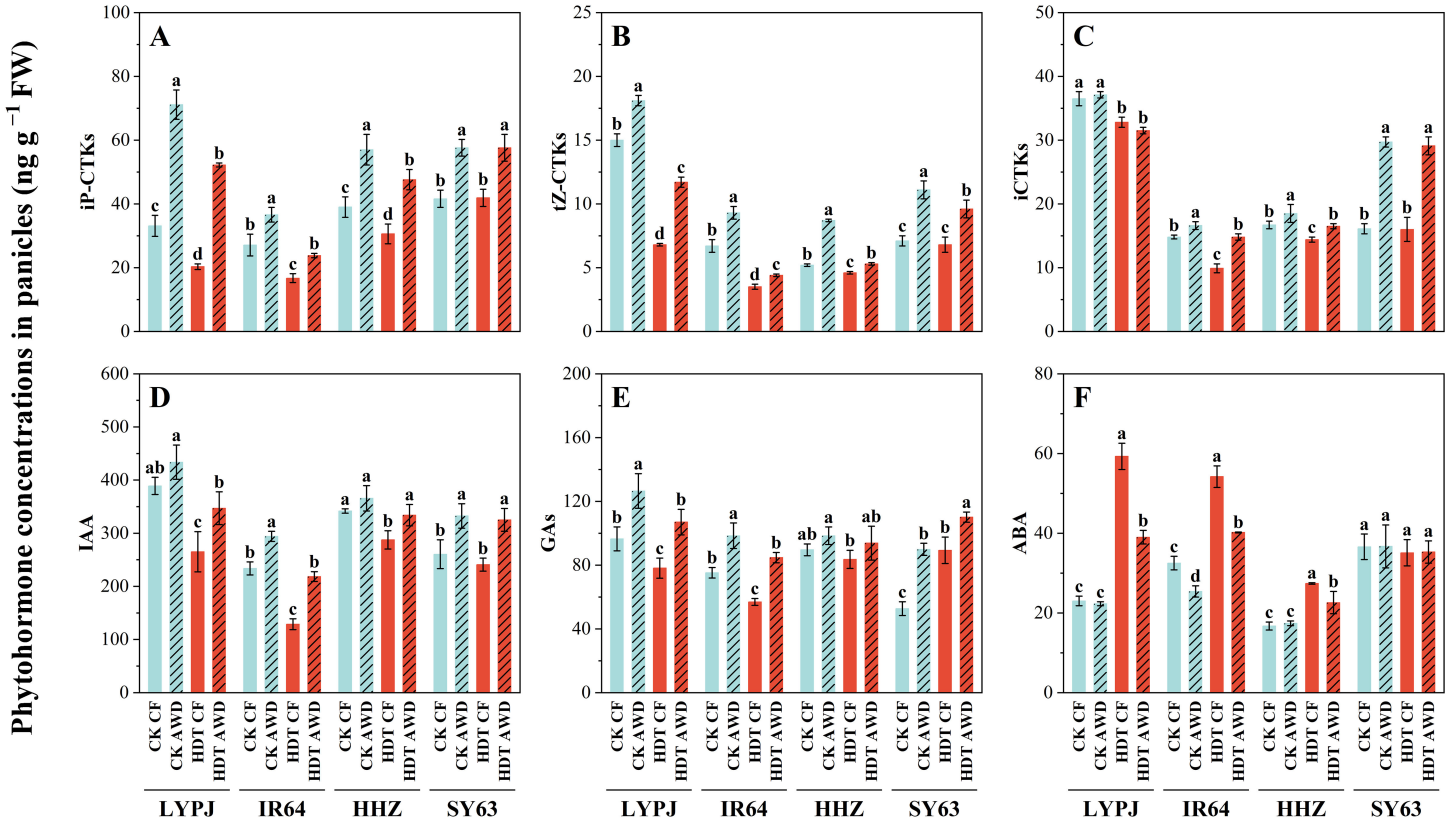
Effects of alternate wetting and drying irrigation at the tillering stage on phytohormone concentrations of iP-CTKs **(A)**, tZ-CTKs **(B)**, iCTKs **(C)**, IAA **(D)**, GAs **(E)**, and ABA **(F)** in panicles under high daytime temperature at the panicle initiation stage. Data are presented as mean ± standard deviation (n = 3). Different letters on the top of columns indicate statistical significant difference among the four treatment combinations across temperature and irrigation treatments for each variety at the *P* < 0.05 level by the least significant difference test. CK, control temperature treatment; HDT, high daytime temperature treatment; CF, continuous flooding; AWD, alternate wetting and drying irrigation. iP-CTKs: content of iPMP, iP, and iPR, tZ-CTKs: content of tZ and tZR, iCTKs: content of inactive cytokinin compounds (tZ9G+iP9G), IAA: content of indole-3-acetic acid, GAs: content of GA1, GA3 and GA4, ABA: content of abscisic acid.

Under CF, HDT obviously decreased the panicle tCTK content in LYPJ, IR64 and HHZ by 29%, 31% and 19%, respectively, while had no significant effect on SY63 (**Figure 4**). The effect of HDT on the tCTK content in four varieties was consistent under AWD and CF, and the effect on iCTKs, tZ-type CTKs, and iP-type CTKs was similar to that on tCTKs. AWD significantly increased the panicle tCTK and aCTK content in all four varieties under HDT and CK. Additionally, it increased the tZ-type CTK and iP-type CTK content in LYPJ, IR64, and HHZ under HDT and CK. AWD showed no significant effect on the iCTK content in LYPJ, but increased that in IR64, HHZ, and SY63 under HDT and CK.

Furthermore, HDT significantly reduced the GA content in LYPJ and IR64, but increased the GA content in SY63 by 69%, while showed no significant effect on HHZ under both CF and AWD. AWD markedly elevated the GA content in LYPJ, IR64, and SY63 under CK and HDT, while had no significant effect on the GA content in HHZ. HDT significantly reduced the IAA content in LYPJ and IR64 under CF, and in LYPJ, IR64, and HHZ under AWD. AWD notably enhanced the panicle IAA content in IR64 and SY63 under CK, and in all four varieties under HDT. HDT significantly increased the panicle ABA content in LYPJ, IR64, and HHZ under both CF and AWD. Additionally, AWD reduced the ABA content in IR64 under CK, and in LYPJ, IR64, and HHZ under HDT.

### 3.8 Effects of alternate wetting and drying irrigation on the expression of genes for cytokinin metabolism in panicles

**Figure 5:**
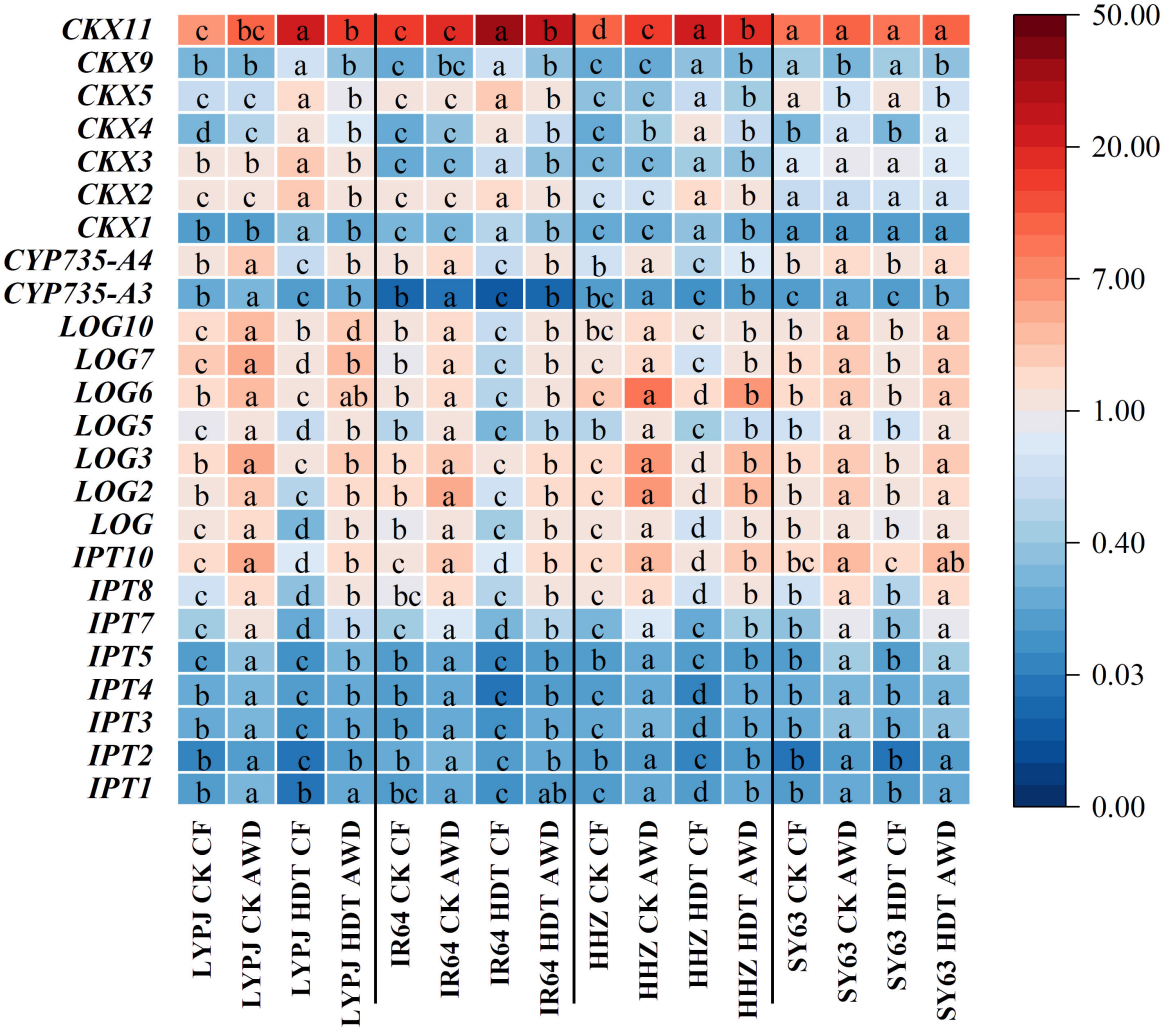
Effects of alternate wetting and drying irrigation at the tillering stage on expression of genes involved in CTK metabolism in panicles under high daytime temperature at the panicle initiation stage. Different letters indicate statistical significant difference among the four combinations across temperature and irrigation treatments for the same gene in the same variety at the *P* < 0.05 level by the least significant difference test. CK, control temperature treatment; HDT, high daytime temperature treatment; CF, continuous flooding; AWD, alternate wetting and drying irrigation. *CKX* gene for CTK oxidase; *IPT* gene for isopentenyl transferase, *CYP735A* gene for cytokinin hydroxylases, and *LOG* gene for CTK-activating enzymes. The expression level of panicle *LOG* gene of LYPJ under CF-CK condition was set as 1, and the relative expression of other genes in panicle was calculated with the expression of this gene as reference.

Under CF, HDT significantly downregulated the expression of *IPT*, *CYP735A*, and *LOG*, while upregulated that of *CKX* in the panicles of LYPJ, IR64, and HHZ (**Figure 5**). In contrast, HDT led to no significant change in the expression of these genes in SY63 under CF. Under AWD, HDT induced similar changing trends in these genes of all varieties, but the magnitude of reduction was smaller than that under CF. Under CK, AWD increased the expression of *IPT*, *CYP735A*, and *LOG* in all varieties. Under HDT, AWD upregulated the expression of *CKX4* and *CKX11* while downregulated that of *CKX5* and *CKX9* of SY63, while most *CKX* genes remained unaffected. Notably, under HDT, AWD maintained its promotive effects on *IPT*, *CYP735*, *LOG* and suppressed *CKX* expression in LYPJ, IR64, and HHZ.

### 3.9 Principal component analysis of plant physiological traits associated with yield, spikelet number, spikelet fertility, and pollen viability under high temperature

**Figure 6:**
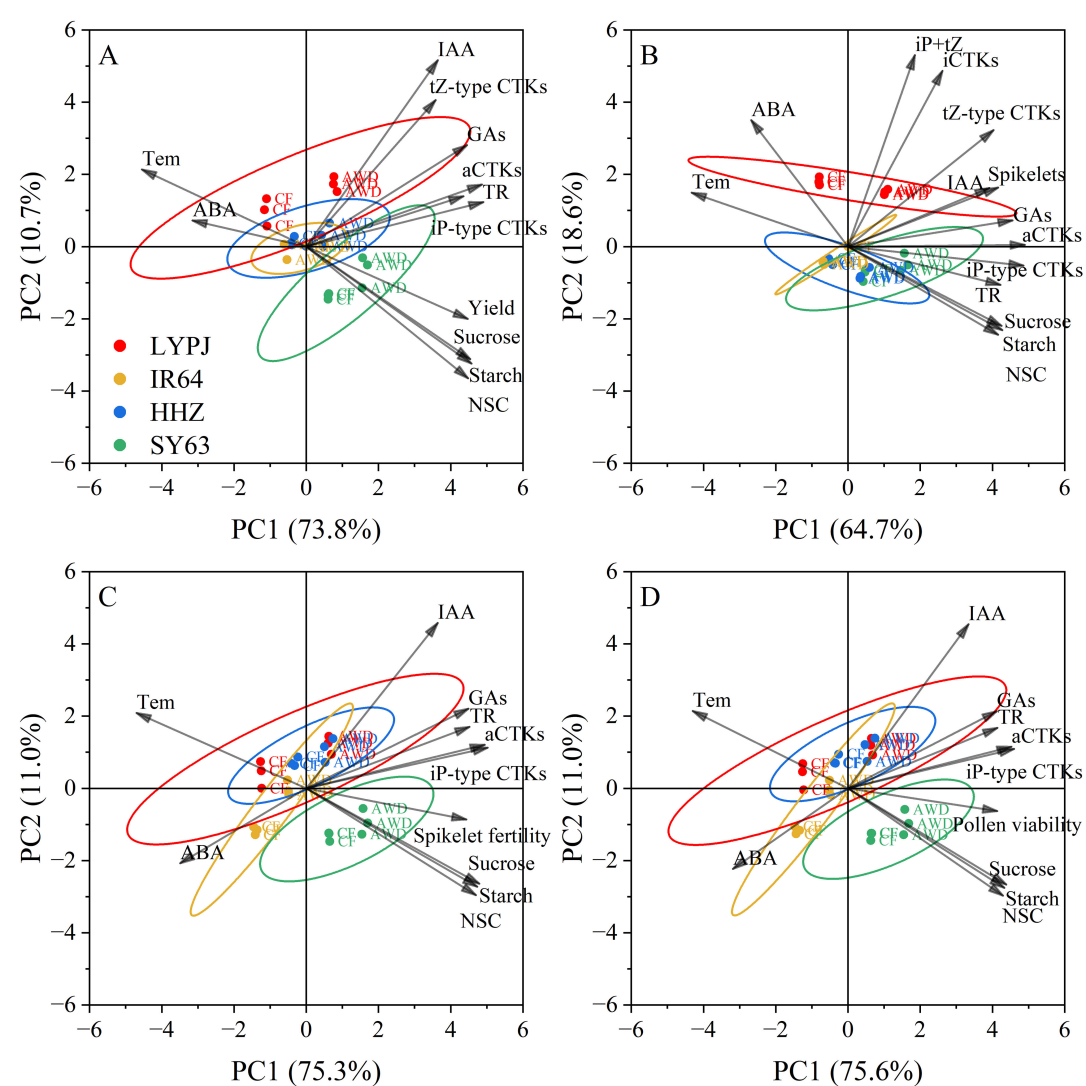
Principal component analysis for the physiological attributes determining yield **(A)**, spikelet number **(B)**, spikelet fertility **(C)** and pollen viability **(D)** under high daytime temperature. The rice varieties LYPJ (red), IR64 (yellow), HHZ (blue) and SY63 (green) are distinguished by color ellipses. TR: leaf transpiration rate, Tem: panicle temperature, NSCs: non-structural carbohydrates content in panicle, Starch: starch content in panicle, Sucrose: sucrose content in panicle, iCTKs: content of biologically inactive CTKs (tZ9G+iP9G) in panicle, aCTKs: content of biologically active CTKs(tZ, tZR, iPMP, iP and iPR) in panicle, tZ-type CTKs: content of tZ and tZR in panicle, iP-type CTKs: content of iPMP, iP, and iPR in panicle, IAA: content of indole-3-acetic acid in panicle, GAs: content of GA_1_, GA_3_ and GA_4_ in panicle, ABA: content of abscisic acid in panicle.

In the PCA of physiological traits related to yield (**Figure 6A**), the first two principal components together accounted for 84.5% of the total variance (PC1: 73.8%; PC2: 10.7%). PC1 showed strong positive loadings for carbohydrate contents (NSCs, starch, sucrose), growth-promoting hormone contents (aCTKs, iP-type CTKs, GAs), and transpiration rate (TR), indicating their primary positive impacts on yield formation. Conversely, PC1 showed significant negative correlations with the panicle temperature (Tem) and ABA, suggesting that an increase in Tem and ABA level markedly suppresses the yield. PC2 was mainly associated with IAA and tZ-type CTKs, reflecting their secondary contribution to the yield.

In terms of spikelet number (**Figure 6B**), PC1 (64.7%) and PC2 (18.6%) explained 83.3% of the total variance. Hormone contents (aCTKs, iP-type CTKs, tZ-type CTKs, IAA, GAs), carbohydrate contents (NSCs, starch, sucrose), and TR dominated the positive loadings of PC1, highlighting their crucial roles in increasing spikelet number. PC2 showed positive loadings for iP+tZ CTKs and iCTKs, indicating that they have some auxiliary regulatory effects on spikelet development. Notably, Tem and ABA level exhibited strong negative loadings on PC1, indicating their inhibitory effects on spikelet initiation under heat stress.

In terms of spikelet fertility (**Figure 6C**) and pollen viability (**Figure 6D**), PC1 and PC2 explained 86.3% and 86.6% of the total variance, respectively. IAA was specifically correlated with positive PC2 values, while Tem and ABA were strongly linked to negative PC1 values. All other physiological traits were clustered within the positive domain of PC1, highlighting their consistent positive roles in reproductive success.

The PCA biplot reveals distinct physiological responses among the four rice varieties. LYPJ (red) under CF was clustered in the PC1-negative quadrant, indicating its sensitivity to HDT stress. Conversely, AWD shifted LYPJ toward the PC1-positive region, indicating that AWD improves its heat tolerance. IR64 (yellow) and HHZ (blue) showed phenotypic stability, and the data points were mainly clustered near the origin. SY63 (green) was consistently clustered in the PC1-positive quadrant under HDT, and had no overlapping confidence ellipse with LYPJ, implying different responses of the two varieties to high temperature.

## 4 Discussion

### 4.1 Impact of high-temperature stress at the panicle initiation stage on yield formation in rice

In this study, HDT at the PI stage reduced the yield of LYPJ, IR64, and HHZ under CF (**Table 1**), which is consistent with the findings of Wu et al. (2016). The yield loss in the three varieties can be mainly attributed to decreases in seed setting rate (by 29% averagely across the three varieties), grain number per panicle (by 18%) under CF. HDT resulted in a significant decline in pollen viability (**Figure 1**), similar to the observed by Wang et al. (2021) and Hu et al. (2021). Pollen viability is often affected by high panicle temperature, ABA content and sugar supply in panicles (Fukuoka et al., 2012; Guan et al., 2023; Hu et al., 2021; Zhao et al., 2023). Our results show that HDT significantly increased the panicle temperature (**Figure 2**) and panicle ABA content in four varieties but the heat tolerant SY63 (**Figure 4**), while reduced the content of assimilates in panicles (**Table 3**), these heat responses led to lower pollen viability with abnormal shape and spikelet fertility (**Figure 1**). Therefore, the decline in seed setting rate under HDT at the PI stage can be primarily ascribed to the decline in spikelet fertility (**Table 1**, **Figure 1**).

**Figure 7:**
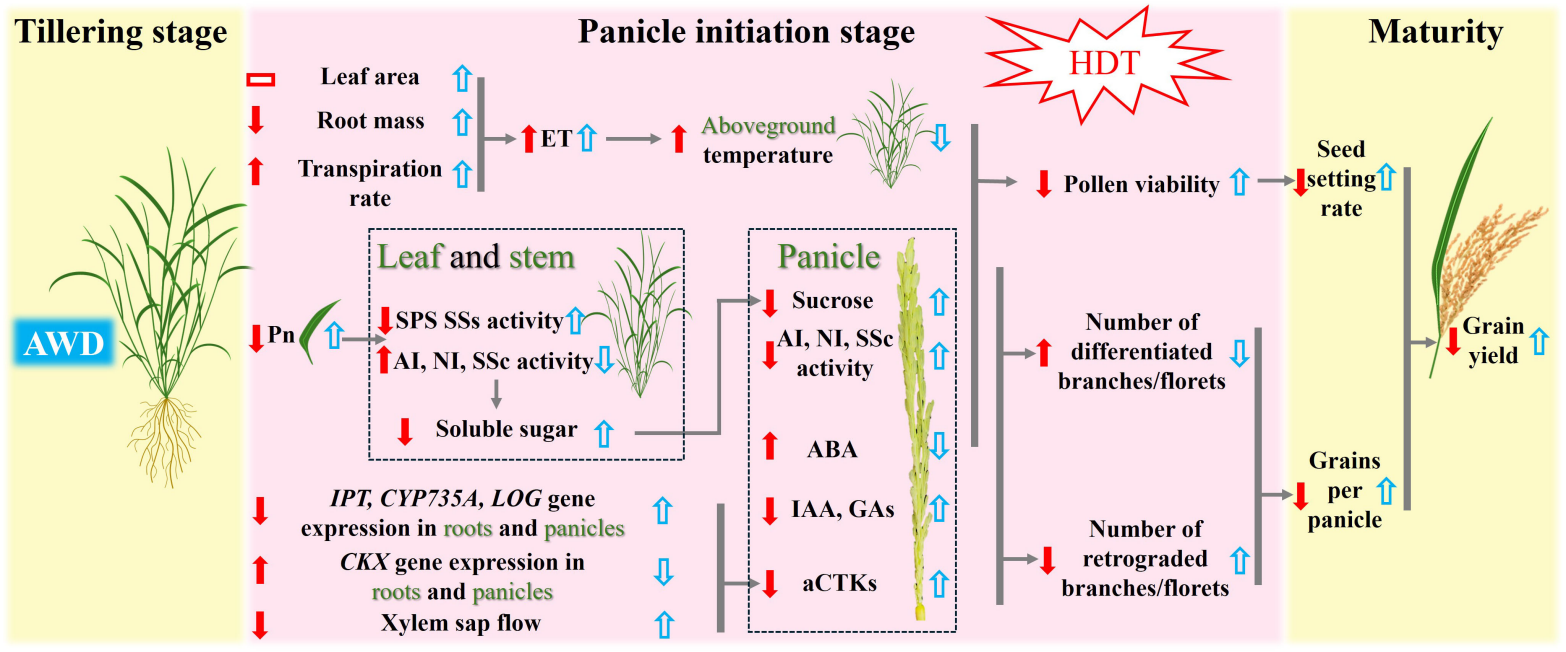
Proposed mechanisms for heat tolerance acquisition at the panicle initiation stage in heat-sensitive rice variety LYPJ via alternate wetting and drying irrigation at the tillering stage. The up and down red arrows indicate up- and down-regulation by high daytime temperature (HDT) under both two irrigation treatments, and the up and down blue arrows indicate up- and down-regulation under high daytime temperature by the alternate wetting and drying irrigation. The red rectangles indicate no significant effect on the given traits by HDT. ET: plant evapotranspiration, Pn: photosynthetic rate, SPS: sucrose phosphate synthase, SSs: sucrose synthase in the synthetic direction, AI: acid invertase, NI: neutral invertase, SSc: Sucrose synthase in the cleavage direction, *CKX* gene encodes CTK oxidase for CTK degradation, *IPT* gene for isopentenyl transferase, *CYP735A* gene for cytokinin hydroxylases, *LOG* gene for CTK-activating enzymes, aCTKs: content of biologically active CTKs(tZ, tZR, iPMP, iP and iPR), IAA: content of indole-3-acetic acid, GAs: content of GA_1_, GA_3_ and GA_4_, ABA: content of abscisic acid.

Our results show that HDT significantly reduced the net photosynthetic rate in LYPJ and IR64 under CF (**Table 3**), which is consistent with the findings of Xiong et al. (2017). Although HDT showed no significant effect on leaf NSCs content, it increased the starch content in leaves and stems of LYPJ, IR64, and HHZ (**Supplementary Table S5**), which may be attributed to less spikelet per panicle and poor grain filling (**Table 2**, **Figure 1**). Importantly, HDT suppressed sucrose synthesis in both leaves and stems, while accelerated sucrose hydrolysis via up-regulating the invertase activity (**Supplementary Table S6**). These changes resulted in increased starch content in source organs, reduced sucrose transport to panicles (**Supplementary Tables S5, S6**), ultimately impairing spikelet development (**Figure 1**, **Table 2**). This finding supports the observation by Chen et al. (2021) that the source-sink sucrose translocation obstacle underlies the heat-induced decrease in spikelet number. Li et al. (2015) demonstrated that reduction in anther sucrose directly led to pollen abortion. Additionally, sucrose content in panicles showed the strongest positive correlation with pollen viability (**Figure 6D**, **Table 3**; **Supplementary Table S4**). This carbohydrate metabolism disruption related to source-sink translocation of sucrose may be one of the mechanisms underlying HDT-induced spikelet loss (**Figure 7**).

Our results show that HDT significantly reduced the number of spikelets per panicle in LYPJ, IR64, and HHZ (**Table 2**). These results are consistent with the report of Wang et al. (2016). In the study, spikelet development was positively regulated by CTKs, GAs, IAA, and carbohydrates, and negatively regulated by ABA (**Figure 6**; **Supplementary Table S4**), the result supports the previous findings (Ding et al., 2014; Wang et al., 2020a; Xu et al., 2020b; Wu et al., 2019). Under CF, HDT significantly suppressed expression of CTK biosynthesis genes in roots and panicles in LYPJ, IR64, and HHZ, while upregulated that of the CTK degradation gene (**Supplementary Figure S2**; **Figure 5**). Additionally, HDT impaired the translocation of CTKs from the roots to the aerial tissues by reducing xylem sap flow (**Supplementary Table S7**). These changes resulted in low levels of aCTKs in panicles (**Figure 4**). These observations align with the results of Wu et al. (2017). These results strongly suggest that the reduction of spikelet differentiation can be attributed to the reduction of aCTKs in panicles (**Figure 7**). Notably, HDT induced the reduction in IAA and GA and high ABA accumulation in both roots and panicles (**Figure 4**; **Supplementary Figure S3**). Spikelet fertility and yield traits were positively correlated with GA and IAA, but negatively correlated with ABA (**Figure 6**; **Supplementary Table S4**). Therefore, these results, together with previous reports, indicate that hormonal homeostasis dysregulation results in the reduction of spikelets under HDT at the PI stage, thereby resulting in substantial yield loss in rice (**Figure 7**).

### 4.2 Mechanisms underlying the enhanced heat tolerance during panicle initiation by the alternate wetting and drying irrigation at the tillering stage in rice

Our results demonstrated that AWD at the tillering stage significantly increased the grain yield under HDT, the increases can be primarily attributed to the coordinated increases in both seed setting rate and grains per panicle (**Table 1**). Similar improvement in grain formation under HDT induced by AWD and mild drought have been reported by Duan et al. (2012) and Feijó et al. (2023). AWD at the tillering stage enhanced the seed setting rate under HDT by significantly improving pollen viability and spikelet fertility (**Figure 1**). Fukuoka et al. (2012) have demonstrated that elevated panicle temperature is a major cause of spikelet sterility, and transpiration can help to reduce the panicle temperature (Wang et al., 2021). Our results indicate that AWD at the tillering stage significantly elevated the root dry weight and xylem sap flow rate at the PI stage (**Supplementary Table S7**), increased leaf area and leaf transpiration rate (**Figure 2**). Similar observations were reported by Ishfaq et al. (2020) and Zhao et al. (2021). The changes responded to AWD collectively mitigate heat injury on yield formation (**Figures 6**, **7**).

Guan et al. (2023) reported that HDT leads to carbohydrate metabolism disorders, resulting in decreases in pollen viability. Our results show that AWD mitigated HDT-induced damage to photosynthetic rate (**Table 3**), increasing sucrose content in leaves and stems and sucrose supply to panicles (**Supplementary Table S5**, **Supplementary Table S6**), which is consistent with the findings of Yang et al. (2017) and Zang et al. (2024). AWD enhanced the allocation of NSCs to panicles under HDT (**Table 3**), as well as increased the activities of AI, NI, and SSc in panicles (**Figure 3**), which is similar to the report of Zang et al. (2024). These changes may promote the breakdown of sucrose in panicles and storage and utilization of carbohydrates, thereby preparing the plant to withstand high temperature by enhancing pollen viability and panicle differentiation (**Figure 7**). Wang et al. (2020b) also reported that high temperature at the PI stage inhibited sucrose breakdown in young panicles, leading to reduced spikelet differentiation and increased spikelet degeneration. Together, these findings suggest that AWD enhances reproductive development under HDT not only by improving pollen viability through optimized carbohydrate metabolism, but also by promoting spikelet differentiation (**Figure 7**).

In this study, AWD at the tillering stage significantly enhanced the differentiation of branches and spikelets while inhibited their degeneration (**Table 2**), which is similar to the observation under AWD applied at the tillering and grain-filling stages reported by Cao et al. (2017). Conversely, Jiang et al. (2021) reported that continuous AWD treatment from the tillering to the grain-filling stages reduced the spikelets per panicle. This discrepancy may be due to differences in the stage of AWD application and the degree of drying. Our results reveals that AWD at the tillering stage optimized hormonal homeostasis through the following mechanisms: (1) upregulating the expression of key CTK biosynthesis genes (*IPT*, *LOG*, *CYP735A*) in roots and panicles, while inhibiting that of the catabolic gene *CKX* (**Figure 5**; **Supplementary Figure S2**); (2) enhancing xylem sap flow to promote the transport of aCTKs to panicles (**Supplementary Table S7**, **Supplementary Figure S3**); (3) coordinately increasing IAA and GA levels in panicles while reducing ABA accumulation (**Figure 4**). These findings are consistent with those of Xu et al. (2018) and Chen et al. (2024). Exogenous application of CTKs, IAA, and GAs significantly promoted spikelet differentiation (Xu et al., 2020b). Additionally, ABA accumulation in anthers under high-temperature conditions led to pollen sterility (Zhao et al., 2023). However, the reduction of root and panicle ABA accumulation after AWD (**Figure 4**; **Supplementary Figure S3**) supports the findings of Abid et al. (2017) and Xu et al. (2018), who noted that drought and AWD decreased the ABA level. Therefore, the increases in CTKs, GAs and IAA contents and decrease in ABA accumulation after AWD improve spikelet differentiation, and the AWD-induced re-balance of plant hormones plays a crucial role in mitigating reduction in grain yield formation under HDT (**Table 2**, **Figures 6**, **7**).

### 4.3 Genotypic variation in mitigating high temperature stress during panicle initiation after alternate wetting and drying irrigation at the tillering stage

The heat-tolerant variety SY63 maintained stable yield and yield components under HDT, the heat-sensitive varieties LYPJ and IR64 displayed severely thermal damage (**Tables 1**, **2**, **Figure 1**). These results are consistent with previous studies of genotype-specific heat responses (Hu et al., 2024; Wu et al., 2019). Interestingly, Yang and Zhang (2010) reported that AWD increased the seed setting rate of LYPJ, thus improving its yield; however, Zhou et al. (2017) found that full-growth-period AWD reduced the yield of SY63. These findings suggest that different rice varieties have different responses to the timing and intensity of AWD. In this study, AWD at the tillering stage increased the yield under HDT by 114% in LYPJ and 60% in IR64, but only by 11% in HHZ and 17% in SY63 (**Table 1**). In LYPJ and IR64, the yield improvement could be mainly ascribed to increased seed setting rate and grain number; in contrast, AWD had little impact on the seed setting rate of HHZ and SY63, but increased the grain number in both varieties, indicating genotype-specific responses to AWD. Therefore, these results suggest that AWD at the tillering stage enhances heat tolerance, especially in heat sensitive varieties (**Figure 7**).

In the study, SY63 exhibited the lowest panicle temperature under HDT, probably due to its higher leaf transpiration rate and evapotranspiration rate of the whole plant (**Figure 2**). This finding is consistent with the transpiration cooling by Xiong et al. (2015) and Tian et al. (2024). After AWD, LYPJ exhibited the largest leaf area and greater increases in transpiration rate and evapotranspiration compared with SY63, accompanying higher decrease of panicle temperature (**Figure 2**), the observation may explain the more increase in grain yield of LYPJ (**Table 1**). Therefore, AWD at the tillering stage enhances transpiration and is beneficial to heat tolerance by acting as a physical cooling, the water management may effectively mitigate heat damage in heat sensitive varieties. Secondly, compared with heat-tolerant varieties HHZ and SY63, heat-sensitive varieties LYPJ and IR64 showed lower leaf sugar supply capacity and lower NSCs in pancles (**Table 3**). However, LYPJ showed the most increase in content of panicle NSCs after AWD, and the most increase in panicle sucrose hydrolase activities under HDT among all varieties (**Figure 3**), mitigating the damage to pollen viability and spikelet differentiation. Liu et al. (2013) observed higher sugar accumulation in the reproductive organs in rice plants exposed to high temperature stress, enhancing the heat resistance. Thirdly, the variety SY63 maintained stable and higher content of aCTKs in panicles due to high synthesis capacity and low catabolism under HDT among all varieties (**Figures 4**, **5**), but LYPJ had the highest proportion of iCTKs under HDT. The findings are similar to the results of Wu et al. (2017). AWD resulted in larger increases in panicle aCTKs of LYPJ relative to SY63. Duan et al. (2012) reported that AWD resulted greater improvement in grain yield of heat sensitive varieties under high temperature. These results collectively suggest that stronger cooling, large increases of carbohydrates and aCTKs in panicles after AWD can explain the genotypic variation in mitigating high temperature stress during panicle initiation, and AWD may alleviate heat damage more effectively in heat-sensitive varieties (**Figure 7**).

## 5 Conclusion

In this study, AWD at the tillering stage was found to alleviate yield reduction caused by HDT during the PI stage in rice by enhancing the seed setting rate and increasing the number of grains per panicle. AWD decreased the panicle temperature under HDT, enhanced leaf photosynthetic rate, facilitated greater sucrose transport to panicles, and promoted its utilization in panicles. These changes collectively improved the pollen viability, leading to higher spikelet fertility and seed setting rate. Additionally, AWD also elevated the aCTK level and their proportion relative to tCTKs in panicles under HDT, as well as the concentrations of IAA and GAs. These hormonal adjustments promoted spikelet differentiation and reduced spikelet degeneration, ultimately increasing the number of spikelets and grains per panicle. Therefore, implementation of AWD at the tillering stage can enhance rice heat tolerance, particularly in heat-sensitive varieties, by improving physiological traits that support reproductive growth under high temperature. Considering convenience and water saving in rice production practice, AWD is a sustainable strategy for mitigation of heat-induced yield loss under global warming. However, the effectiveness of AWD largely depends on several factors, including regional thermal conditions and precipitation, the timing and intensity of water deficit, and varietal sensitivity to drought. Therefore, the successful implementation of AWD requires site-specific adjustments to ensure yield stability.

## Data Availability

The original contributions presented in the study are included in the article/**Supplementary Material**. Further inquiries can be directed to the corresponding author.
