# Supplementary material for: Alternate wetting and drying irrigation at tillering stage enhances the heat tolerance of rice by increasing sucrose and cytokinin content in panicles
Source: Front Plant Sci. 2025 May 29;16:1598652. doi: 10.3389/fpls.2025.1598652 (PMC12159064; doi:10.3389/fpls.2025.1598652)
Supplement: Supplementary file 1 [file DataSheet1.docx]

Supplementary Material

# Supplementary Tables

**Supplementary Table 1.** Temperature settings in greenhouse during the temperature treatment

| Time (h) | CK (℃) | HDT (℃) |
| --- | --- | --- |
| 0:00-7:00 | 26 | 26 |
| 7:00-9:00 | 27 | 30 |
| 9:00-10:00 | 29 | 33 |
| 10:00-11:00 | 31 | 35 |
| 11:00-15:00 | 32 | 37 |
| 15:00-16:00 | 31 | 35 |
| 16:00-17:00 | 29 | 33 |
| 17:00-19:00 | 27 | 30 |
| 19:00-24:00 | 26 | 26 |

**Supplementary Table 2.** Program for elution in hormone determination

| Running time  (min) | Solvent A Conc. (%) | Solvent B Conc. (%) |
| --- | --- | --- |
| 1.0 | 90 | 10 |
| 3.0 | 80 | 20 |
| 4.0 | 70 | 30 |
| 5.0 | 60 | 40 |
| 5.1 | 20 | 80 |
| 6.0 | 10 | 90 |
| 7.0 | 10 | 90 |
| 7.1 | 90 | 10 |
| 10.0 | STOP | STOP |

Solvent A:10 mM ammonium acetate in solution, solvent B: HPLC-grade acetonitrile

**Supplementary Table 3.** Primers used for qPCR in this study

| Gene | Accession number (NCBI) | Forward primer 5′-3′ | Reverse primer 5′-3′ | References |
| --- | --- | --- | --- | --- |
| *OsIPT1* | XM_015774823 | ACCAAGCCCAAGGTTATCTTCGTGC | TCGTCGGTGACCTTGTTGGTGATGA | Ding et al., 2014 |
| *OsIPT2* | XM_015774814 | AGTCACCCAAGCCCAAGGTCGTCTT | CTCCTCGGTGACCTTGTTCGTGATG | Ding et al., 2014 |
| *OsIPT3* | XM_015783380 | GAGCTGTGCTTCCTGTGGGTGGACT | GCGACCTTGTACTTGTCTCCGTGCG | Ding et al., 2014 |
| *OsIPT4* | XM_015777162 | TGGATGTGGTGACGAACAAGGTGAC | GATCTACGTCGACCCAGAGGAAGCA | Ding et al., 2014 |
| *OsIPT5* | XM_015790849 | AGGTGATCAACGCCGACAAGCTGCA | TCGACGAGCTCCTCGATGTAGGAGT | Ding et al., 2014 |
| *OsIPT7* | XM_015782976 | TGGACGACATGGTGGACGCTGGCAT | GCTTTGATGTCGTCGATCGCCTCGG | Ding et al., 2014 |
| *OsIPT8* | XM_026020134 | GTCGACGACGATGTTCTCGACGAAT | TGTTGGCCTTGATCTCGTCTATCGC | Ding et al., 2014 |
| *OsIPT10* | XM_015788701 | TCTCAATCACCACCGTCGTCT | AGTTCATGCCAGGACTCAAGC | Gao et al., 2019 |
| *OsLOG* | NM_001406413 | TTGCACTGCCTGGCGGCTAC | CGAGCTAGGGGCCGCCTTTG | Ding et al., 2014 |
| *OsLOGL2* | XM_015767376 | AGCGCACAGAAAAGAGAAGC | GGCATGAGTGCTTTTGGAAT | Ding et al., 2014 |
| *OsLOGL3* | NM_001423909 | GTGCTGCATTGTCTGCAGTT | GGTCATGAGAGTCTTGGGGA | Ding et al., 2014 |
| *OsLOGL5* | NM_001402329 | CTCTGCAAGCTCGAGGAATAC | GAGTAGATGCTTCCGGCG | Wang et al., 2020 |
| *OsLOGL6* | NM_001419668 | GTGGGATCTCTCTCCATCCA | TGCCTCCCTCCATTGTAGAC | Ding et al., 2014 |
| *OsLOGL7* | NM_001420857 | ATGGAACAATGGAGGAGCTG | AGCAAGGGAAAACAAAAGCA | Ding et al., 2014 |
| *OsLOGL10* | NM_001423062 | AGCTAAGGGCAGAGGAGAGG | CATATCAGCAACTGGCCTCA | Ding et al., 2014 |
| *OsCYP735A3* | XM_015794046 | TCACTGGGCAATGTCAAAGACTCCC | AGGAGAGGTGTTCTTGGCCTTGTTC | Ohashi et al., 2017 |
| *OsCYP735A4* | XM_015755276 | GTTCGTTTTGGCGCCATAGCTAGAG | CGCACCATATTACCAGCCGGGCCAC | Ohashi et al., 2017 |
| *OsCKX1* | NM_001401472 | ACAAGGCGTACCTGGCGCAC | TGGCCAGGGGAGAGCAGCTT | Ding et al., 2014 |
| *OsCKX2* | NM_001406314 | CCCATGAACCGCAACAAGTGGGACA | ACTCTGCCTGGCTGCCGTAGT | Ding et al., 2014 |
| *OsCKX3* | NM_001423067 | TCGTGCCAAGCGCTGGGATT | CCTGGCCCGAGGATGCACCT | Ding et al., 2014 |
| *OsCKX4* | NM_001398017 | GCCACAGGACCCAGTGCAGG | TTCAGCCACGGGTGTGGGACT | Ding et al., 2014 |
| *OsCKX5* | NM_001401734 | CGCTGCTGGGCGAGCTGAAT | CGCCTTGTGCACGCGGTCTA | Ding et al., 2014 |
| *OsCKX9* | NM_001420459 | GCCAGGATTCCTCTTGAACCTGC | ACGCACTGGGTCCTGCGGAT | Ding et al., 2014 |
| *OsCKX11* | NM_001422591 | CAACGCAATCATTGACGCC | TTGCACCCCTCCCAAATGT | Zhang et al., 2020 |
| Actin | AB047313 | ATGAAGATCAAGGTGGTCGC | GATCTCAGCCTTGGCAATCC | Liu et al., 2023 |

**References**

1. Ding, C., You, J., Chen, L., Wang, S., Ding, Y., (2014). Nitrogen fertilizer increases spikelet number per panicle by enhancing cytokinin synthesis in rice. *Plant Cell Rep.* 33, 363–371. https://doi.org/10.1007/s00299-013-1536-9.
2. Gao, S., Xiao, Y., Xu, F., Gao, X., Cao, S., Zhang, F., Wang, G., Sanders, D., Chu, C., (2019). Cytokinin‐dependent regulatory module underlies the maintenance of zinc nutrition in rice. *New Phytologist.* 224, 202–215. https://doi.org/10.1111/nph.15962
3. Liu, L., Cui, K., Qi, X., Wu, Y., Huang, J., Peng, S., (2023). Varietal responses of root characteristics to low nitrogen application explain the differing nitrogen uptake and grain yield in two rice varieties. *Front. Plant Sci.* 14, 1244281. https://doi.org/10.3389/fpls.2023.1244281.
4. Ohashi, M., Ishiyama, K., Kojima, S., Kojima, M., Sakakibara, H., Yamaya, T., Hayakawa, T., (2017). Lack of cytosolic glutamine synthetase1;2 activity reduces nitrogen-dependent biosynthesis of cytokinin required for axillary bud outgrowth in rice seedlings. *Plant and Cell Physiology.* 58, 679–690. https://doi.org/10.1093/pcp/pcx022
5. Wang, Q., Zhu, Y., Zou, X., Li, F., Zhang, J., Kang, Z., Li, X., Yin, C., Lin, Y., (2020). Nitrogen deficiency-induced decrease in cytokinins content promotes rice seminal root growth by promoting root meristem cell proliferation and cell elongation. *Cells.* 9, 916. https://doi.org/10.3390/cells9040916
6. Zhang, W., Peng, K., Cui, F., Wang, D., Zhao, J., Zhang, Y., Yu, N., Wang, Yuyang, Zeng, D., Wang, Yonghong, Cheng, Z., Zhang, K., (2021). Cytokinin oxidase/dehydrogenase OsCKX11 coordinates source and sink relationship in rice by simultaneous regulation of leaf senescence and grain number. *Plant Biotechnology Journal.* 19, 335–350. https://doi.org/10.1111/pbi.13467

**Supplementary Table 4.** Pearson correlation of yield, spikelet number per panicle, spikelet fertility and pollen viability with plant physiological traits under high temperature treatment

| Item | Yield | Spikelets  per panicle | Spikelet  fertility | Pollen  viability |
| --- | --- | --- | --- | --- |
| Transpiration rate | 0.677*** | 0.577** | 0.643*** | 0.663*** |
| Panicle temperature | -0.787*** | -0.518** | -0.725*** | -0.742*** |
| Panicle NSCs content | 0.849*** | 0.545** | 0.811*** | 0.817*** |
| Panicle starch content | 0.812*** | 0.535** | 0.776*** | 0.787*** |
| Panicle Sucrose content | 0.847*** | 0.496* | 0.816*** | 0.822*** |
| Panicle contents of active CTKs | 0.797*** | 0.838*** | 0.700*** | 0.725*** |
| Panicle contents of inactive CTKs | ns | 0.623** | ns | ns |
| Panicle content of tZ-type CTKs | 0.423* | 0.742*** | ns | ns |
| Panicle content of iP-type CTKs | 0.833*** | 0.822*** | 0.755*** | 0.776*** |
| Panicle content of iP + tZ | ns | 0.468* | ns | ns |
| Panicle content of GAs | 0.710*** | 0.780*** | 0.590** | 0.631*** |
| Panicle content of IAA | 0.584** | 0.885*** | 0.469* | 0.492* |
| Panicle content of ABA | -0.781*** | -0.482* | -0.851*** | -0.841*** |

n = 24 across the four varieties, two irrigation treatments, and three replicates. ***, **, *: represents significance at 0.001, 0.01, and 0.05 level, ns: regression not significance.

**Supplementary Table 5.** Effects of alternate wetting and drying irrigation at the tillering stage on sugar contents in leaves and stems under high temperature at the panicle initiation stage

| Variety | Temperature | Irrigation | Leaf |  |  |  | Stem |  |  |  |
| --- | --- | --- | --- | --- | --- | --- | --- | --- | --- | --- |
|  |  |  | NSCs | Soluble sugar | Starch | Sucrose | NSCs | Soluble sugar | Starch | Sucrose |
|  |  |  | mg g^−1^ | mg g^−1^ | mg g^−1^ | mg g^−1^ | mg g^−1^ | mg g^−1^ | mg g^−1^ | mg g^−1^ |
| LYPJ | CK | CF | 61.6±2.4 b | 50.3±2.0 b | 11.4±1.1 c | 28.3±0.4 b | 178.5±2.1 b | 99.4±0.6 b | 79.2±2.3 bc | 66.3±3.3 b |
|  |  | AWD | 67.6±1.7 a | 55.7±1.4 a | 11.9±0.3 c | 31.7±1.7 a | 193.8±1.2 a | 108.8±2.3 a | 85.0±3.5 b | 73.5±1.3 a |
|  | HDT | CF | 64.3±1.2 ab | 46.1±1.0 c | 18.2±0.6 a | 24.3±0.3 c | 162.9±6.0 c | 69.5±1.3 d | 93.4±4.8 a | 38.5±1.3 c |
|  |  | AWD | 63.9±1.5 b | 49.2±1.2 b | 14.8±0.7 b | 31.0±0.7 a | 181.1±3.8 b | 103.5±1.8 c | 77.6±2.1 c | 65.8±2.3 b |
| IR64 | CK | CF | 58.5±1.5 ab | 47.5±0.6 b | 11.0±1.0 b | 23.6±0.9 b | 157.3±2.6 b | 88.1±2.6 ab | 69.2±0.7 c | 52.8±2.7 b |
|  |  | AWD | 60.2±1.1 a | 50.0±0.9 a | 10.2±0.7 b | 26.5±0.7 a | 164.2±1.9 a | 93.1±1.0 a | 71.1±0.9 bc | 58.4±2.1 a |
|  | HDT | CF | 56.3±1.8 b | 41.0±0.9 d | 15.3±1.0 a | 21.4±0.6 c | 148.7±4.1 c | 70.2±2.5 c | 78.5±1.6 a | 39.3±1.7 c |
|  |  | AWD | 58.7±2.2 ab | 44.4±1.1 c | 14.3±1.0 a | 24.7±1.7 ab | 158.5±2.7 ab | 85.5±3.5 b | 73.0±1.5 b | 51.6±1.2 b |
| HHZ | CK | CF | 65.3±1.6 a | 54.5±0.7 b | 10.7±1.0 b | 30.6±0.9 ab | 172.8±4.9 ab | 98.8±3.6 a | 74.0±2.0 a | 62.7±1.8 b |
|  |  | AWD | 67.7±1.8 a | 57.3±1.4 a | 10.4±0.4 b | 32.9±0.6 a | 181.3±1.8 a | 104.6±2.3 a | 76.7±1.0 a | 67.2±0.8 a |
|  | HDT | CF | 67.4±1.0 a | 51.8±1.7 b | 15.6±0.7 a | 29.5±1.3 b | 166.8±5.6 b | 89.9±3.0 b | 76.9±2.6 a | 54.4±1.0 c |
|  |  | AWD | 67.6±1.6 a | 52.9±1.5 b | 14.6±0.6 a | 32.0±1.8 a | 175.5±4.1 ab | 98.9±3.2 a | 76.6±1.0 a | 61.8±2.1 b |
| SY63 | CK | CF | 68.2±2.2 c | 56.4±1.9 b | 11.8±0.3 c | 35.8±1.0 bc | 208.4±7.1 b | 124.2±6.4 b | 84.2±1.0 c | 79.5±3.3 c |
|  |  | AWD | 74.9±1.1 a | 62.2±1.4 a | 12.7±0.3 b | 42.5±2.4 a | 238.5±6.2 a | 141.4±4.8 a | 97.0±1.8 a | 98.1±6.0 b |
|  | HDT | CF | 70.3±1.4 bc | 56.3±1.0 b | 14.1±0.4 a | 34.1±2.2 c | 207.5±5.7 b | 125.3±4.3 b | 82.3±1.7 c | 83.4±4.0 c |
|  |  | AWD | 72.8±1.1 ab | 60.4±1.6 a | 12.4±0.6 bc | 40.2±3.0 ab | 237.6±5.0 a | 146.5±3.5 a | 91.1±1.5 b | 106.7±3.1 a |

Data are presented as mean ± standard deviation (n = 3). Different letters within an identical column indicate statistical significant difference among the four treatment combinations across temperature and irrigation treatments for each variety at the *P* < 0.05 level by the least significant difference test. NSCs: non-structural carbohydrates, CK: control temperature treatment, HDT: high daytime temperature treatment, CF: continuous flooding, AWD: alternate wetting and drying irrigation.

**Supplementary Table 6**. Effects of alternate wetting and drying irrigation at the tillering stage on activities of sucrose metabolism enzymes in leaves and stems under high temperature at the panicle initiation stage

| Variety | Temperature | Irrigation | Leaf |  |  |  |  | Stem |  |  |  |  |
| --- | --- | --- | --- | --- | --- | --- | --- | --- | --- | --- | --- | --- |
|  |  |  | SPS | SSs | AI | NI | SSc | SPS | SSs | AI | NI | SSc |
|  |  |  | μmol  mg^−1^ h^−1^ | μmol  mg^−1^ h^−1^ | μmol  mg^−1^ h^−1^ | μmol  mg^−1^ h^−1^ | μmol  mg^−1^ h^−1^ | μmol  mg^−1^ h^−1^ | μmol  mg^−1^ h^−1^ | μmol  mg^−1^ h^−1^ | μmol  mg^−1^ h^−1^ | μmol  mg^−1^ h^−1^ |
| LYPJ | CK | CF | 7.0±0.2 c | 8.1±0.3 c | 2.3±0.2 b | 1.2±0.0 b | 0.3±0.0 b | 3.0±0.2 b | 3.5±0.3 b | 3.9±0.2 c | 1.0±0.0 d | 3.1±0.0 b |
|  |  | AWD | 8.0±0.2 a | 9.7±0.3 a | 2.3±0.2 b | 1.1±0.0 b | 0.3±0.0 b | 3.8±0.1 a | 4.1±0.1 a | 4.2±0.2 c | 1.4±0.2 c | 2.6±0.1 c |
|  | HDT | CF | 6.2±0.1 d | 6.3±0.4 d | 3.4±0.2 a | 1.6±0.0 a | 0.7±0.1 a | 1.5±0.1 c | 2.3±0.3 c | 6.8±0.5 a | 2.5±0.1 a | 5.5±0.1 a |
|  |  | AWD | 7.5±0.3 b | 8.8±0.1 b | 2.3±0.1 b | 1.2±0.1 b | 0.3±0.0 b | 2.9±0.1 b | 3.4±0.1 b | 5.0±0.1 b | 2.2±0.2 b | 3.5±0.4 b |
| IR64 | CK | CF | 7.0±0.0 b | 7.6±0.0 b | 5.8±0.2 c | 2.3±0.1 ab | 1.4±0.1 a | 2.7±0.3 a | 10.0±0.4 b | 5.1±0.3 b | 1.9±0.0 c | 2.6±0.2 b |
|  |  | AWD | 7.6±0.2 a | 8.3±0.3 a | 6.1±0.1 bc | 2.1±0.0 c | 1.5±0.1 a | 3.0±0.0 a | 10.9±0.5 a | 3.5±0.1 c | 1.9±0.1 c | 3.0±0.2 b |
|  | HDT | CF | 5.8±0.1 d | 5.9±0.3 d | 6.9±0.3 a | 2.4±0.1 a | 1.7±0.2 a | 2.0±0.1 b | 7.2±0.6 d | 8.6±0.4 a | 4.2±0.2 a | 4.2±0.4 a |
|  |  | AWD | 6.6±0.1 c | 6.9±0.2 c | 6.3±0.2 b | 2.1±0.1 bc | 1.5±0.1 a | 2.6±0.1 a | 8.9±0.3 c | 5.4±0.2 b | 2.7±0.1 b | 3.9±0.3 a |
| HHZ | CK | CF | 7.0±0.3 b | 7.8±0.3 b | 5.2±0.1 ab | 1.5±0.1 a | 0.9±0.0 b | 3.0±0.2 ab | 10.9±0.3 a | 5.3±0.2 b | 2.3±0.1 bc | 2.4±0.1 c |
|  |  | AWD | 8.1±0.4 a | 8.6±0.0 a | 4.8±0.3 b | 1.6±0.1 a | 0.9±0.1 b | 3.4±0.4 a | 11.6±0.3 a | 4.9±0.1 c | 2.1±0.1 c | 2.3±0.1 c |
|  | HDT | CF | 6.5±0.1 b | 7.0±0.1 c | 5.7±0.2 a | 1.5±0.1 a | 1.0±0.1 a | 2.6±0.2 b | 9.6±0.8 b | 6.0±0.2 a | 3.3±0.2 a | 3.2±0.1 a |
|  |  | AWD | 7.7±0.1 a | 7.9±0.1 b | 5.0±0.3 b | 1.5±0.1 a | 0.9±0.1 ab | 3.0±0.2 ab | 10.6±0.5 ab | 5.1±0.1 bc | 2.4±0.1 b | 2.6±0.1 b |
| SY63 | CK | CF | 8.0±0.3 b | 9.8±0.1 b | 2.4±0.1 a | 0.9±0.1 a | 0.7±0.1 b | 3.4±0.3 c | 3.7±0.2 b | 4.2±0.1 b | 1.7±0.1 b | 3.3±0.2 ab |
|  |  | AWD | 10.1±0.3 a | 11.6±0.3 a | 2.0±0.1 b | 0.9±0.1 a | 0.9±0.0 a | 4.2±0.3 b | 4.8±0.1 a | 4.7±0.4 a | 2.4±0.1 a | 3.1±0.1 bc |
|  | HDT | CF | 7.9±0.5 b | 9.7±0.3 b | 2.5±0.2 a | 1.0±0.1 a | 0.9±0.1 a | 3.9±0.3 bc | 3.9±0.3 b | 4.6±0.3 a | 2.2±0.2 a | 3.5±0.2 a |
|  |  | AWD | 9.5±0.3 a | 11.2±0.4 a | 1.8±0.1 b | 0.9±0.1 a | 0.9±0.0 a | 4.9±0.3 a | 5.0±0.4 a | 4.5±0.2 a | 2.4±0.1 a | 2.9±0.2 c |

Data are presented as mean ± standard deviation (n = 3). Different letters within an identical column indicate statistical significant difference among the four treatment combinations across temperature and irrigation treatments for each variety at the *P* < 0.05 level by the least significant difference test. CK: control temperature treatment, HDT: high daytime temperature treatment, CF: continuous flooding, AWD: alternate wetting and drying irrigation. SPS: sucrose phosphate synthase, SSs: sucrose synthase in the synthetic direction, AI: acid invertase, NI: neutral invertase, SSc: Sucrose synthase in the cleavage direction.

**Supplementary Table 7.** Effects of alternate wetting and drying irrigation at the tillering stage on dry matter and xylem sap flow at panicle initiation stage under high temperature at the panicle initiation stage

| Variety | Temperature | Irrigation | Stem | Leaf | Root | Shoot | R/S ratio | Xylem sap flow |
| --- | --- | --- | --- | --- | --- | --- | --- | --- |
|  |  |  | g plant^−1^ | g plant^−1^ | g plant^−1^ | g plant^−1^ | % | g plant^−1^ h^−1^ |
| LYPJ | CK | CF | 17.9±1.0 c | 13.2±0.7 c | 16.5±0.7 c | 32.4±1.7 c | 0.5±0.0 b | 1.0±0.0 c |
|  |  | AWD | 24.0±2.1 ab | 15.2±1.6 ab | 30.3±2.8 a | 40.8±3.6 ab | 0.7±0.0 a | 1.3±0.0 a |
|  | HDT | CF | 19.7±1.7 bc | 15.0±1.3 bc | 12.0±0.4 d | 35.4±2.7 bc | 0.3±0.0 c | 0.8±0.1 d |
|  |  | AWD | 25.6±3.2 a | 16.1±0.7 a | 23.2±0.9 b | 42.7±3.9 a | 0.5±0.0 b | 1.2±0.0 b |
| IR64 | CK | CF | 16.6±0.2 b | 11.6±0.7 c | 13.7±0.3 c | 29.4±0.8 c | 0.5±0.0 ab | 0.8±0.0 c |
|  |  | AWD | 20.5±1.5 a | 13.9±0.7 b | 17.9±1.3 a | 35.8±2.0 b | 0.5±0.0 a | 1.3±0.1 a |
|  | HDT | CF | 16.8±1.2 b | 11.3±0.5 c | 12.2±0.4 d | 29.0±1.7 c | 0.4±0.0 bc | 0.7±0.1 d |
|  |  | AWD | 22.4±0.5 a | 15.7±0.5 a | 15.5±0.6 b | 39.3±0.9 a | 0.4±0.0 c | 1.0±0.0 b |
| HHZ | CK | CF | 17.2±0.8 c | 11.0±0.8 bc | 9.9±1.1 b | 28.7±1.5 b | 0.3±0.0 b | 0.9±0.1 b |
|  |  | AWD | 21.8±1.1 a | 12.5±0.4 a | 16.2±0.1 a | 35.0±1.2 a | 0.5±0.0 a | 1.4±0.1 a |
|  | HDT | CF | 16.9±1.0 c | 7.4±0.9 c | 7.3±0.9 c | 24.6±2.0 c | 0.3±0.0 b | 0.8±0.0 b |
|  |  | AWD | 19.1±0.5 b | 12.5±0.6 a | 14.3±1.6 a | 32.2±1.0 a | 0.4±0.1 a | 1.3±0.1 a |
| SY63 | CK | CF | 19.6±1.3 a | 11.3±0.2 bc | 14.3±2.1 b | 32.4±1.4 ab | 0.4±0.0 b | 1.0±0.0 b |
|  |  | AWD | 19.8±0.6 a | 13.6±0.6 a | 21.8±1.0 a | 35.3±0.6 a | 0.6±0.0 a | 1.2±0.1 a |
|  | HDT | CF | 18.1±1.2 a | 11.2±1.0 c | 13.4±1.3 b | 30.7±2.1 b | 0.4±0.0 b | 0.9±0.1 b |
|  |  | AWD | 18.6±1.5 a | 12.7±0.8 ab | 19.4±1.0 a | 33.0±2.3 ab | 0.6±0.1 a | 1.2±0.0 a |

Data are presented as mean ± standard deviation (n = 3). Different letters within an identical column indicate statistical significant difference among the four treatment combinations across temperature and irrigation treatments for each variety at the *P* < 0.05 level by the least significant difference test. CK: control temperature treatment, HDT: high daytime temperature treatment, CF: continuous flooding, AWD: alternate wetting and drying irrigation, R/S ratio: root/shoot ratio.

# Supplementary Figures


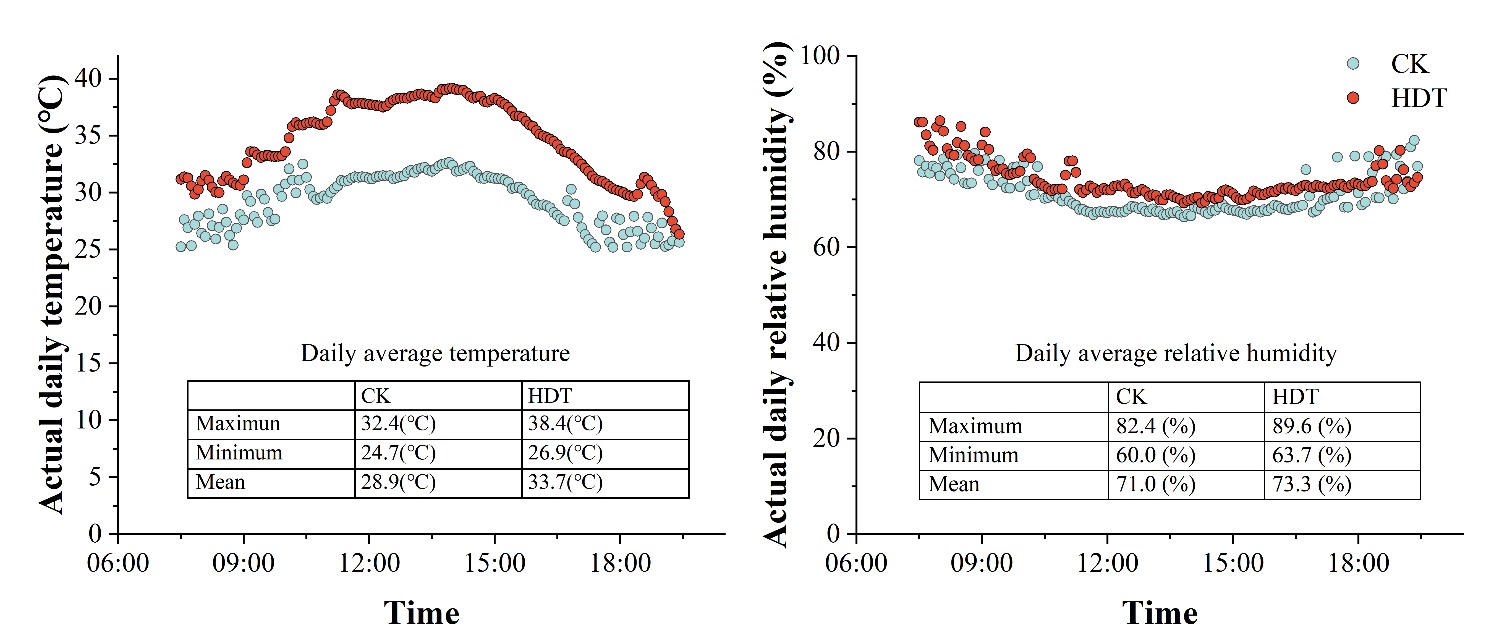
**Supplementary Figure 1.** Actual mean daily temperature and mean relative humidity across 15 days for high-temperature treatment

**
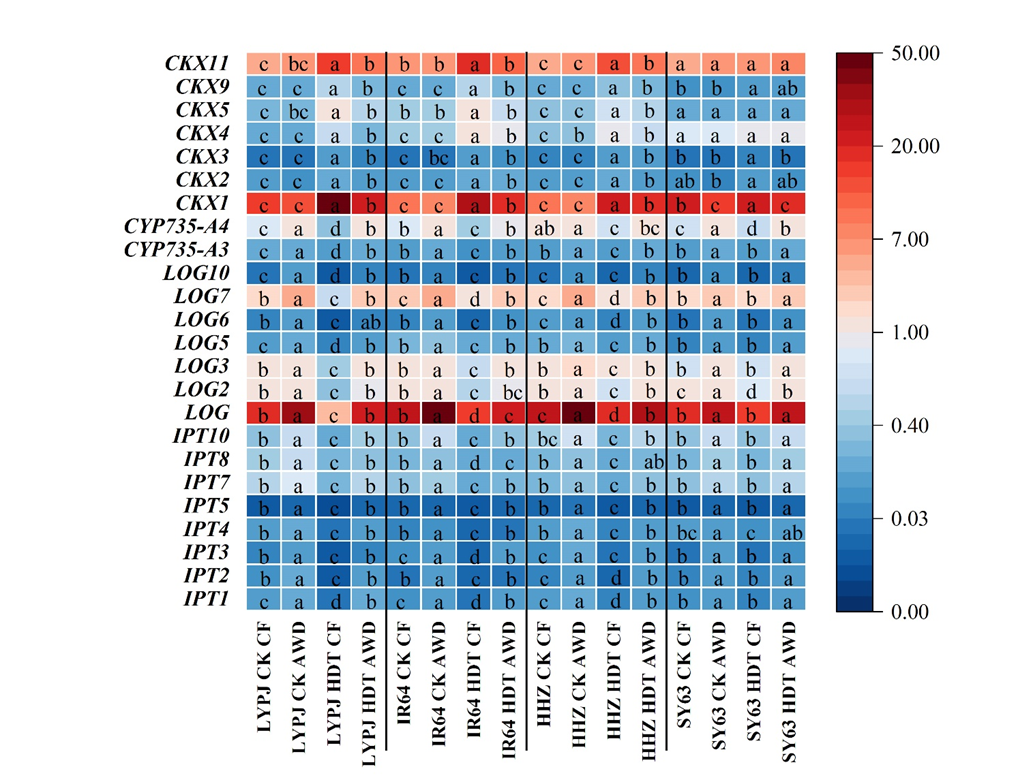
**

**Supplementary Figure 2.** Effects of alternate wetting and drying irrigation at the tillering stage on expression of genes involved in CTK metabolism in roots under high temperature at the panicle initiation stage.

Different letters indicate statistical significant difference among the four combinations across temperature and irrigation treatments for the same gene in the same variety at the *P* < 0.05 level by the least significant difference test. CK: control temperature treatment, HDT: high daytime temperature treatment, CF: continuous flooding, AWD: alternate wetting and drying irrigation. *CKX* gene for CTK oxidase, *IPT* gene for isopentenyl transferase, *CYP735A* gene for cytokinin hydroxylases, and *LOG* gene for CTK-activating enzymes. The expression level of root *LOG2* gene of LYPJ under CF-CK condition was set as 1, and the relative expression of other genes in roots was calculated with the expression of this gene as reference.


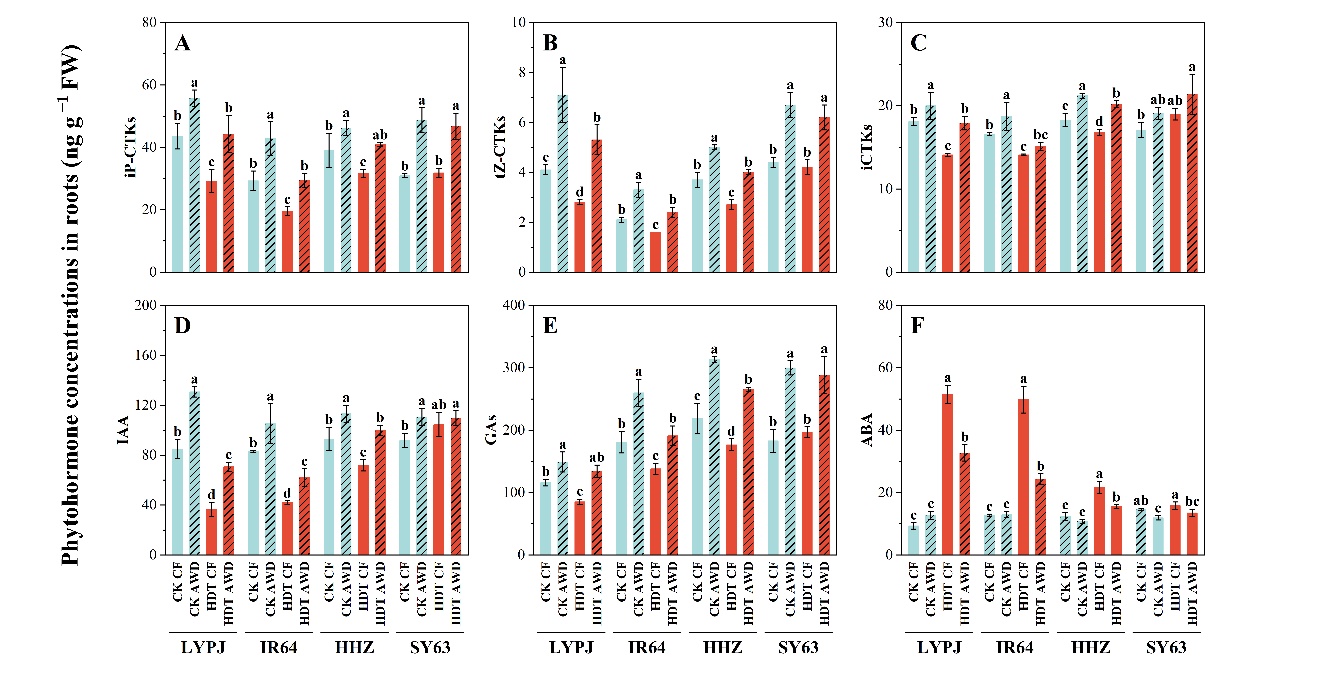


**Supplementary Figure 3.** Effects of alternate wetting and drying irrigation at the tillering stage on phytohormone concentrations of iP-CTKs (A), tZ-CTKs (B), iCTKs (C), IAA (D), GAs (E), and ABA (F) in roots under high temperature at the panicle initiation stage.

Data are presented as mean ± standard deviation (n = 3). Different letters on the top of columns indicate statistical significant difference among the four treatment combinations across temperature and irrigation treatments for each variety at the *P* < 0.05 level by the least significant difference test. CK: control temperature treatment, HDT: high daytime temperature treatment, CF: continuous flooding, AWD: alternate wetting and drying irrigation. iP-CTKs: content of iPMP, iP, and iPR, tZ-CTKs: content of tZ and tZR, iCTKs: content of inactive cytokinin compounds (tZ9G+iP9G), IAA: content of indole-3-acetic acid, GAs: content of GA_1_, GA_3_ and GA_4_, ABA: content of abscisic acid.
